# Use of non-specific immunoglobulins in Catalonia in three third-level hospitals: a descriptive analysis of a hospital-prescribed medication registry

**DOI:** 10.3389/fphar.2024.1420682

**Published:** 2024-12-16

**Authors:** J. Riera-Arnau, E. Ballarín, R. Llop, E. Montané, P. Hereu, G. Vancells, N. Padullés-Zamora, A. M. Barriocanal, G. Cardona-Peitx, C. Casasnovas, J. B. Montoro, M. Nuñez, E. Santacana Juncosa, A. Selva-O’Callaghan, X. Solanich, M. Sabaté Gallego

**Affiliations:** ^1^ Department of Clinical Pharmacology, Vall d’Hebron Hospital Universitari, Vall Hebron Institut de Recerca (VHIR), Vall d’Hebron Barcelona Hospital Campus, Barcelona, Spain; ^2^ Department of Pharmacology, Therapeutics and Toxicology, Universitat Autònoma de Barcelona (UAB), Barcelona, Spain; ^3^ Department of Clinical Pharmacology, Hospital Universitari de Bellvitge, Bellvitge Hospital Campus, Bellvitge Biomedical Research Institute (IDIBELL), Barcelona, Spain; ^4^ Patology and Experimental Therapeutics Department, Universitat de Barcelona (UB), Barcelona, Spain; ^5^ Department of Clinical Pharmacology, Hospital Universitari Germans Trias i Pujol, Germans Trias i Pujol Hospital Campus, Barcelona, Spain; ^6^ Pharmacy Service, Vall d’Hebron Hospital Universitari, Vall d’Hebron Barcelona Hospital Campus, Barcelona, Spain; ^7^ Pharmacy Service, Hospital Universitari de Bellvitge, Bellvitge Hospital Campus, Barcelona, Spain; ^8^ Pharmacotherapy, Pharmacogenetics and Pharmaceutical Technology Group, Bellvitge Biomedical Research Institute (IDIBELL), Barcelona, Spain; ^9^ Fundació d’Investigació en Ciències de la Salut Germans Trias i Pujol (IGTP), Barcelona, Spain; ^10^ Pharmacy Service, Hospital Universitari Germans Trias i Pujol, Barcelona, Spain; ^11^ Neuromuscular Unit, Neurology Department, Bellvitge University Hospital, Barcelona, Spain; ^12^ Neurometabolic Diseases Group, Bellvitge Biomedical Research Institute (IDIBELL) and CIBERER, Barcelona, Spain; ^13^ Internal Medicine Department, Autoimmune Systemic Diseases Unit, Vall d’Hebron Hospital Universitari, Vall d’Hebron Barcelona Hospital Campus, Barcelona, Spain; ^14^ Internal Medicine Department, Hospital Universitari de Bellvitge, Barcelona, Spain; ^15^ Systemic Diseases and Aging Group, Bellvitge Biomedical Research Institute (IDIBELL), Barcelona, Spain; ^16^ Faculty of Medicine and Health Sciences, Universitat de Barcelona, Barcelona, Spain

**Keywords:** non-specific human immunoglobulins, drug utilization, patient safety, hospital registry, discontinuation, costs

## Abstract

**Background:**

The increasing use of non-specific immunoglobulins (NSIGs) and their current shortage show a need for NSIGs’ use prioritization. Data from a clinical perspective are necessary, mainly for pediatric patients.

**Objectives:**

The aim of the study was to assess the level of clinical evidence (LoE) of the indications that NSIGs are used for, the reasons for discontinuation, and the costs invested.

**Methods:**

A retrospective multicentric study was conducted on NSIG incident users between September 2019 and December 2021 retrieved from the Registry of Patients and Treatments (RPT) from Catalonia (Spain). LoE was categorized as A) authorized indications, B) unauthorized with scientific support, C) unauthorized without support, and D) unknown (UNK), following local and the United Kingdom’s guidelines as a sensitivity analysis. We also estimated overall spending and costs per patient visit.

**Results:**

A total of 400 patients were included (17.3% pediatric), with a mean follow-up of 122.1/person-years for adults. The most frequent indications were nervous system and blood diseases. Almost all pediatric patients (56; 81.2%) were treated under A-level indications, as for 217 (65.6%) adults. In the sensitivity analysis, the A-level usage rate decreased to one-third and the B-level usage rate increased by 2–3 times. Furthermore, 37.8% (151) of individuals discontinued. This was predominantly due to remission or no response. The total costs were 868,462.6€/year, with median spending per visit amounting to 1,500€ for adults and 700€ for pediatric patients.

**Conclusion:**

NSIGs are used in clinical practice mainly for approved indications; however, non-approved indications are still an important issue. This could represent a significant economic burden on the healthcare system, focusing on the pediatric population and those at risk for discontinuation with alternative therapeutic options.

## 1 Introduction

Non-specific immunoglobulins (NSIGs) are used for a variety of indications. Some of the approved indications include primary immunodeficiency disorders, chronic inflammatory demyelinating polyradiculoneuropathy (CIDP), and Kawasaki disease. Immunoglobulins can also be used off-label for other conditions based on clinical experience and published cases or small studies. Ruiz-Antorán et al. (2010) showed that 60% of the patients were prescribed NSIGs for authorized indications in our setting, while 40% received them for unauthorized (off-label) indications (Ruiz-Antorán et al., 2010). As the number of both increases, the global demand for immunoglobulins increases by about 6%–8% per year (European Medicines Agency, 2016; Farrugia and Poulis, 2001; N’kaoua et al., 2022; Touraille and Brosch, 2016; So-Osman et al., 2024).

In the past few years, intermittent shortages due to limited supply of plasma have become increasingly frequent (European Medicines Agency, 2016; N’kaoua et al., 2022; Touraille and Brosch, 2016; Spanish agency for medicines and health products, 2021; Immunodeficiency United Kingdom, 2023; So-Osman et al., 2024). Such shortages also affected Catalonia (Spain) due to not only their broad spectrum of indications or manufacturing limitations but also recent critical disease outbreaks (Wörner et al., 2021). Moreover, NSIGs have been used to combat respiratory infections caused by SARS-CoV-2, SARS-CoV, or MERS-CoV (Ruiz-Antorán et al., 2010; Stanworth et al., 2020; AminJafari and Ghasemi, 2020; Vallejo Rodríguez et al., 1999). Adding to the 20% decrease in donations, the price of NSIGs has increased significantly, and the agencies have had to make a supply management plan (Spanish agency for medicines and health products, 2021; Immunodeficiency United Kingdom, 2023).

Some healthcare providers and medical systems have taken steps to optimize the limited supplies of immunoglobulins for patients (World Health Organization, 2022; Canadian Blood Service, 2021; Derman et al., 2021a; Toumi and Urbinati, 2015). Some strategies have been established for NSIG use, like lowering doses, delaying treatments, prioritizing based on medical need, and using alternative therapies where those exist (European Parliament, 2020; Castle and Robertson, 2019; Albin and Cunningham-Rundles, 2014). Furthermore, other studies in our setting (Solís-Díez et al., 2022) have suggested a rationalization plan describing Catalan spending but created a set of prioritization categories based on pharmacy and non-European guidelines (National Blood Authority Australia, 2022; Alberta Ministry of Health Shared Health Manitoba and Saskatchewan Ministry of Health, 2018) instead of the Public Catalan Health System (SISCAT) guidelines. However, both adult and pediatric patients risk not receiving the treatment they need (Stanworth et al., 2020). Hence, it becomes paramount to shape the investments intended for indications with or without scientific evidence, what the cost of each patient means for the healthcare system, and the reasons for discontinuation of NSIGs to elucidate whether the investment has been worthwhile. There can be several reasons for interrupting a treatment with NSIGs, including side effects, inefficacy, or remission of the underlying disease (Nobile-Orazio et al., 2012; Angelotti et al., 2020). Therapy with immunoglobulins is generally considered a safe treatment. Nonetheless, mild-to-severe adverse effects have been reported in 11%–81% of patients (Wittstock et al., 2003).

In our setting, NSIGs are dispensed by the hospital for out- and in-patient use. Thus, medical registries play a key role in the systematic collection of data to obtain valuable information for decision-making support (Solís-Díez et al., 2022; Condino-Neto et al., 2014). In 2014, the Registry of Patients and Treatments (RPT) was implemented in Catalonia by health professionals from SISCAT to monitor the use of high-cost drugs under routine clinical practice conditions (Roig Izquierdo et al., 2020).

Thus, in order to promote safe and rational use of immunoglobulin products, we aim to describe their patterns of use in adult and pediatric patients, and its related expenses. Furthermore, we assess the level of evidence of their indications and the reasons that led to discontinuation of the therapy.

## 2 Materials and methods

### 2.1 Study population and design

**FIGURE 1:**
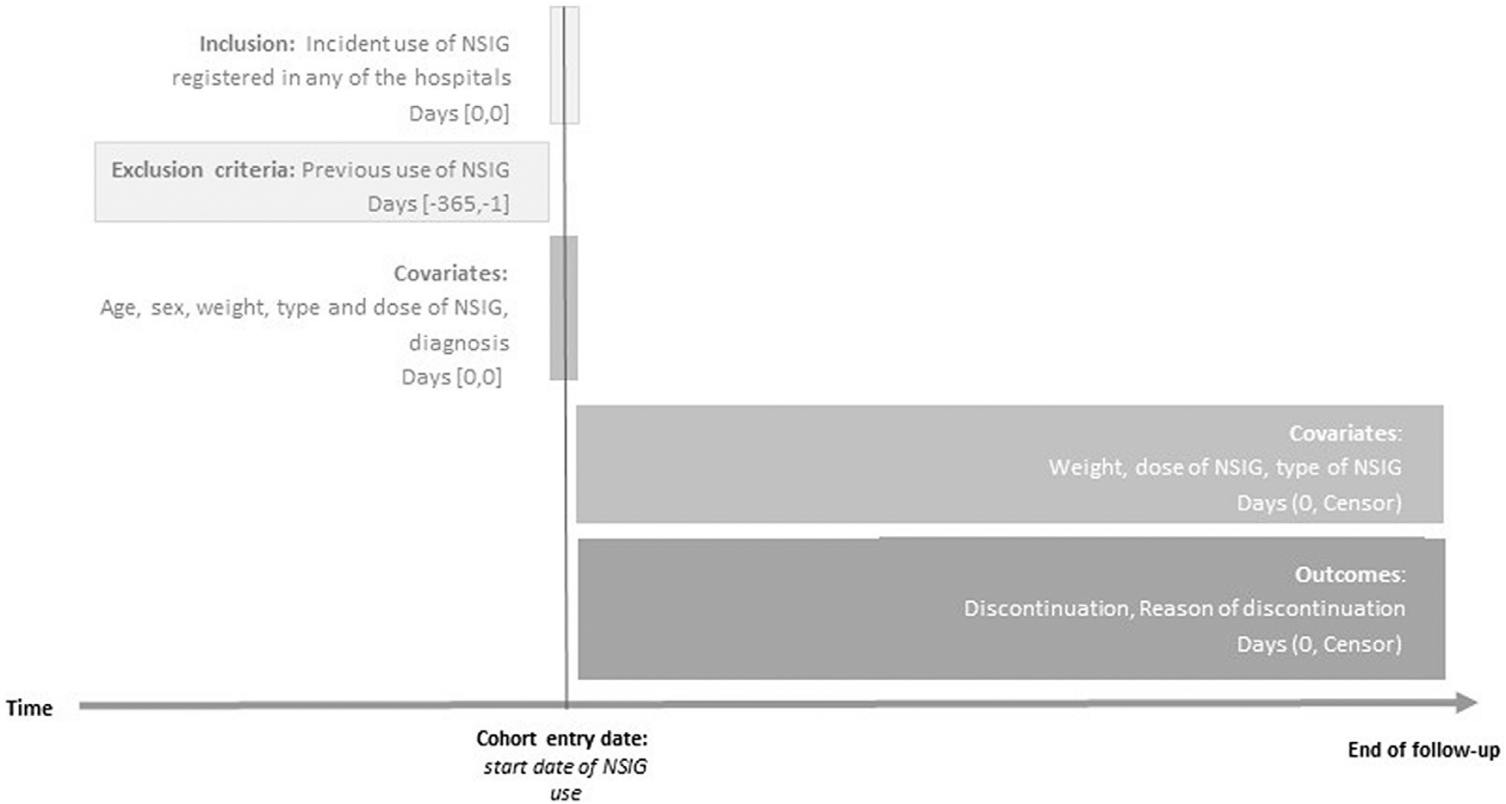
Diagram of the study design. Index date was defined as the date of start of use of non-specific immunoglobulins. End of follow-up (censor) for all outcomes in patients was the earliest date of outcome, discontinuation, death, or 31 December 2021. NSIG, non-specific immunoglobulin.

We conducted an observational cohort multicentric study in three of the biggest third-level hospitals in Catalonia, Spain. We included all new NSIG users between 1 September 2019 and 31 December 2021. The follow-up ended at the time of treatment discontinuation, death, loss of follow-up, or end of the study period (see Figure 1).

### 2.2 Data source and setting

We used the information from third-level hospitals belonging to SISCAT: Vall d’Hebron University Hospital (VHUH), Germans Trias i Pujol University Hospital (GTiPUH), and Bellvitge University Hospital (BUH). These are three of the biggest hospitals included in the Catalan public healthcare system. Only the BUH does not have pediatrics and obstetrics department (Vall D’Hebron Barcelona Hospital Campus, 2024; Bellvitge Hospital Universitari, 2024; Hospital Universitari Germans Trias i Pujol, 2024).

Data were retrieved from the RPT, which is a registry of patients using high-cost drugs under routine clinical practice conditions. From 1 September 2019, it was made mandatory to register NSIG users’ variables, as defined by the Pharmacotherapeutic Harmonization Program (PHF). The start of use of an NSIG and subsequent visits for its administration trigger a record in the RPT. Thus, we can assume that each visit means a dose administration. The general structure of the registry consists of information on basic demographic data, the identification of the treatment, indication, the reason for discontinuation, and other relevant clinical variables at follow-up (Roig Izquierdo et al., 2020).

### 2.3 Exposure

The exposures of interest were either intravascular NSIGs (IVIGs; ATC code J06BA02) or subcutaneous NSIGs (SCIGs; ATC code J06BA10). New users were defined as those with no NSIG prescription or dispensation in the year prior to the cohort entry date (see Figure 1). Thus, a patient might have multiple-incident episodes within the study period. Treatment discontinuation was defined as NSIG stoppage for a year or longer after the last administration date. If a patient changed from one type of NSIG to another in less than 1 year, he/she was considered to be a switcher.

### 2.4 Outcomes

The main outcomes of interest were the level of evidence of the indications for NSIG use, the reasons for discontinuation of the therapy, and the costs invested per visit.

The indications of use are reported using the ICD-10 vocabulary and categorized depending on the level of evidence available for each following the clinical guidelines. As a reference, we applied the SISCAT guideline (Public Catalan Health System, 2021), which is in force in Catalonia. We conducted a sensitivity analysis using the United Kingdom NSIGs guideline because it also focuses on clinical evidence, regardless of economic conditions (Department of Health and Social Care of the Government of United Kingdom, 2011). Other guidelines outside the European setting actually exist but are centered on the prioritization of use in situations of shortage, rather than on evidence of clinical efficacy (Agenzia Italiana del Farmaco, 2022; Agence Nationale de sécurité du médicament et des produits de santé, 2019). The levels of evidence were described as follows: A) indications authorized by the European Medicines Agency (EMA), B) unauthorized indications with substantial scientific support, C) unauthorized indications without sufficient scientific support, and D) UNK: indications with unknown evidence or unclassified (see Supplementary Table S1).

The reasons for discontinuation were established as the PHF requires, i.e., death, remission of the underlying disease, no response to the therapy, drug–drug interaction, toxicity, and patient’s decision, among others (Public Catalan Health System, 2021).

The costs were calculated using the actual purchase price (in euros, €) agreed between the hospitals and the manufacturer, per gram of NSIG, per type (IVIG or SCIG), and year (see Supplementary Table S2). Thus, we took into account the direct costs of drug purchase but not other indirect costs. We estimated the absolute spending and also the costs per patient visit. To calculate the absolute costs per patient, the costs of all patient visits (NSIG administrations) were summed, and then, the median and range were calculated. Note that the result will be time-dependent (the more visits, the more expenditure). To calculate the costs generated per visit, the cost of each visit for each patient was taken into account, and then, the median and range were calculated. Thus, the result is independent of the treatment length. We incorporated in our calculation weight and dosage variations (e.g., induction vs. maintenance dosage) during the follow-up.

### 2.5 Covariates

We extracted information on age, sex assigned at birth, weight, induction dosage, and number of visits and NSIG administrations. Moreover, weight and dosage changes at each follow-up visit were available (see Figure 1). We report age as a categorical variable to distinguish pediatric (<18 years) and adult patients (≥18 years).

### 2.6 Statistical analysis

Descriptive characteristics are given as mean (standard deviation), median (interquartile range or range), or number (percentage). Time of contribution is calculated in person-years. Outcome counts and proportions are presented stratified by age band, sex assigned at birth, the level of evidence, and hospital center. All analyses were conducted using RStudio version 4.1.2 (RStudio Team, 2020).

A sensitivity analysis was performed replicating our calculations of main outcomes using the United Kingdom guidelines reporting NSIG evidence of clinical efficacy. These guidelines also differentiate the level of evidence by the duration of the treatment per indication. As SISCAT guidelines do not differentiate between chronic- and acute-duration usage, we chose the highest piece of evidence in order to compare them (Public Catalan Health System, 2021; Department of Health and Social Care of the Government of United Kingdom, 2011).

### 2.7 Data management and quality control

The de-identification process was performed locally using scripts hosted in a private GitHub repository. Afterward, anonymized data were shared for a centralized analysis in the VHUH.

We performed quality checking procedures on the de-identified data. This included information on missingness, duplicates, inconsistencies, and counts and distributions of variables to screen for outliers. Referees from each participant center reviewed and corrected the issues flagged in the quality report.

No imputation was needed due to the absence of missing data.

### 2.8 Ethical approval

This study was approved by the Comité de Ética de la Investigación con medicamentos (CEIm) of VHUH on 14 February 2022 [protocol reference: VDH-IGG-2021-03; review code: EOM(AG)017/2021(5812)]. Although the centralized approval applies, the CEIm from the BUH and GTiPUH validated the protocol approval (review codes EOMNA001/22 and PI-22-046, respectively). This study was registered under reference EUPAS48635.

The study has been carried out in accordance with the principles from the Declaration of Helsinki and according to current legal regulations (Real Decreto 957/2020). All information obtained in the study has been treated confidentially, in compliance with the Ley Orgánica de Protección de Datos de Carácter Personal (LOPD) 3/2018.

## 3 Results

### 3.1 General characteristics

**FIGURE 2:**
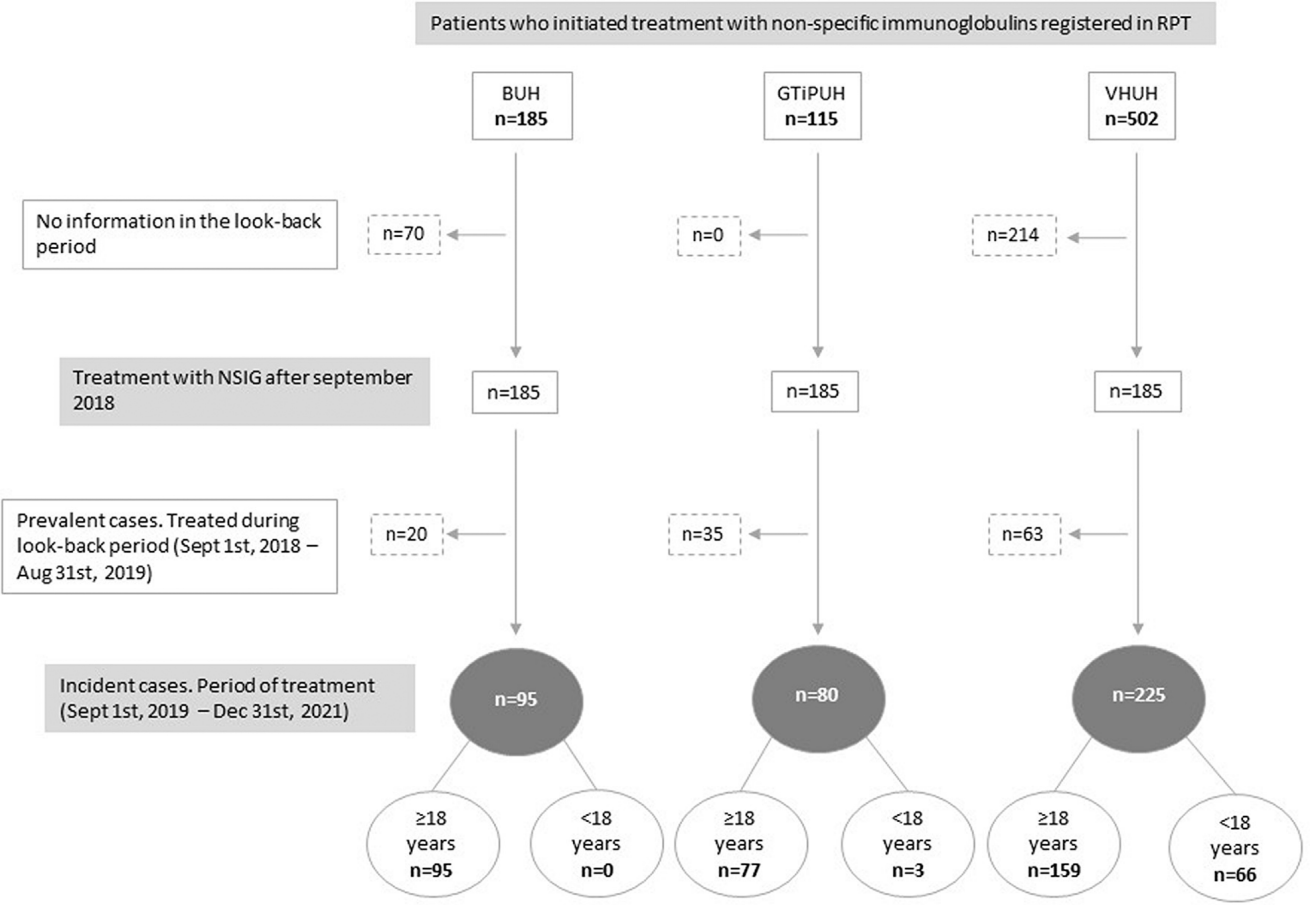
Flowchart of the selection of patients by hospital. BUH, Bellvitge University Hospital; GTiPUH, Germans Trias i Pujol University Hospital; NSIG, Non-specific immunoglobulins; RPT, Registry of Patients and Treatments; VHUH, Vall d'Hebron University Hospital.

This study comprised a total of 400 patients [80 (20.0%) from the GTiPUH, 95 (23.8%) from the BUH, and 225 (56.3%) from the VHUH], 201 (50.3%) of which were female individuals. Pediatric patients (<18 years of age) represented 17.3% (n = 69) (see Figure 2). Across hospital centers, the mean–time contribution to the cohort was similar for adults (mean of 122.1 person-years) and varied substantially for pediatric patients, ranging from 1.44 in GTiPUH to 45.1 person-years in VHUH. In addition, 95.7% (66) of pediatric patients was detected in VHUH. For adult patients in GTiPUH, the use of SCIGs at the start of treatment was approximately 3.5-fold higher than that in the other centers. However, the median dose at baseline was similar between the three hospitals (see Table 1). Overall, we detected a total of two patients with multiple incident episodes during the study period and four switchers. These were counted only once based on the initial treatment.

**TABLE 1:** Baseline characteristics of the study cohort by hospital and age band.

		BUH^b^	GTiPUH	VHUH
		Adults (≥18 years of age)		
Population	All, n (%)	95 (28.7)	77 (23.3)	159 (48.0)
	Woman, n (%)	51 (53.7)	37 (48.1)	88 (55.4)
	Age, median [IQR] years	56.5 [45.8–72.0]	59 [45.0–70.0]	54 [40.0–64.0]
	Weight, median [IQR] kg	69.5 [60.0–78.5]	70 [64.0–80.0]	67 [60.0–75.0]
	Time of follow-up, person-years	106.3	105.4	149.7
	Number of visits, median [range]	2 [1–6]	2 [1–6]	2 [1–7]
Exposure^a^	Intravenous immunoglobulin, n (%)	87 (91.6)	54 (70.1)	147 (92.5)
	Subcutaneous immunoglobulin, n (%)	8 (8.4)	23 (29.9)	12 (7.6)
	Dose at start, median [IQR] g/kg	0.6 [0.4–2.0]	0.8 [0.4–2.0]	0.5 [0.4–2.0]
		Pediatrics (<18 years of age)		
Population	All, n (%)	0	3 (4.3)	66 (95.7)
	Woman, n (%)	**—**	1 (33.3)	24 (36.4)
	Age, median [IQR] years	**—**	7 (5.5–8.5)	9.5 [5.0–13.0]
	Weight, median [IQR] kg	**—**	21 (20.5–21.0)	29.5 [17.8–42.8]
	Time of follow-up, person-years	**—**	1.44	45.1
	Number of visits, median [range]	**—**	1 [1–1]	1 [1–7]
Exposure^a^	Intravenous immunoglobulin, n (%)	**—**	2 (66.7)	63 (95.5)
	Subcutaneous immunoglobulin, n (%)	**—**	1 (33.3)	3 (4.5)
	Dose at start, median [IQR] g/kg	**—**	0.8 [0.6–0.9]	0.4 [0.4–1.0]

BUH, Bellvitge University Hospital; GTiPUH, Germans Trias i Pujol University Hospital; IQR, interquartile range; NA, not applicable; VHUH, Vall d’Hebron University Hospital.

^a^ At the start of follow-up.

^b^ BUH only has an adult population.

The most frequent indications of the use of NSIGs per hospital were diseases of the nervous system in the BUH (53/95; 55.8%) and immunodeficiency replenishment therapy, blood and blood-forming organ diseases in the GTiPUH [32/77 (41.6%) adults and 3/3 (100%) pediatrics] and VHUH [77/159 (48.4%) adults and 19/66 (28.8%) pediatrics] (see Table 2; Supplementary Table S3 for the counts segregated by indication). Discrete differences were detected when stratifying by sex assigned at birth, i.e., slightly more women were being treated for B-level indications than men and the opposite for A-level (see Supplementary Table S4).

**TABLE 2:** Indications of use of non-specific immunoglobulins by hospital, age band, and the level of clinical evidence according to SISCAT guidelines.

Indication	BUH^a^, n (%)	GTiPUH, n (%)		VHUH, n (%)		Total
	≥18 year	≥18 year	<18 year	≥18 year	<18 year	n (%)
A—Authorized						
Immunodeficiency replenishment therapy, and blood and blood-forming organs	18 (19.0)	27 (35.1)	3 (100)	50 (31.3)	15 (22.6)	113 (41.4)
Common variable immunodeficiency	7 (7.4)	15 (19.5)	—	22 (13.8)	3 (4.5)	47 (17.2)
Primary antibody immunodeficiency syndrome (SIP)	5 (5.3)	3 (3.9)	—	9 (5.6)	2 (3.0)	19 (7.0)
Purpura and other hemorrhagic conditions, including idiopathic and immune thrombocytopenia	2 (2.1)	—	2 (66.7)	6 (3.8)	7 (10.6)	17 (6.2)
Chronic lymphatic leukemia with severe secondary hypogammaglobulinemia and recurrent infections	2 (2.1)	2 (2.6)	—	5 (3.1)	—	9 (3.3)
IgG subclass deficiency with recurrent infections	—	5 (6.5)	—	4 (2.5)	—	9 (3.3)
Congenital hypogammaglobulinemias	2 (2.1)	-	1 (33.3)	2 (1.3)	1 (1.5)	6 (2.2)
Combined severe immunodeficiency	—	1 (1.3)	—	—	2 (3.0)	3 (1.1)
Multiple myeloma with severe secondary hypogammaglobulinemia	—	1 (1.3)	—	1 (0.6)	—	2 (0.7)
Nonfamilial hypogammaglobulinemia	—	—	—	1 (0.6)	—	1 (0.4)
Nervous system	43 (45.3)	12 (15.6)	0 (0)	28 (17.5)	1 (1.5)	84 (30.8)
Myasthenia gravis	19 (20.0)	7 (9.1)	—	12 (7.5)	—	38 (13.9)
Chronic inflammatory demyelinating polyneuropathy	8 (8.4)	5 (6.5)	—	12 (7.5)	1 (1.5)	26 (9.5)
Multifocal motor neuropathy	14 (14.7)	—	—	3 (1.9)	—	17 (6.2)
Guillain Barré syndrome	1 (1.1)	—	—	—	—	1 (0.4)
Other inflammatory polyneuropathies	1 (1.1)	—	—	—	—	1 (0.4)
Myasthenia gravis without (acute) exacerbation	—	—	—	1 (0.6)	—	1 (0.4)
Transplantation	7 (7.4)	11 (14.3)	0 (0)	21 (13.2)	37 (56.0)	76 (27.9)
Antibody-mediated rejection treatment in solid organ transplant	7 (7.4)	9 (11.7)	—	4 (2.5)	8 (12.1)	28 (10.3)
Replenishment therapy for allogeneic hematopoietic stem cell transplantation with hypogammaglobulinemia	—	1 (1.3)	—	5 (3.1)	19 (28.8)	25 (9.2)
Kidney transplant	—	1 (1.3)	—	7 (4.4)	4 (6.1)	12 (4.4)
Desensitizer treatment before a high-risk immunological solid organ transplant	—	—	—	2 (1.3)	2 (3.0)	4 (1.5)
Bone marrow transplant	—	—	—	1 (0.6)	2 (3.0)	3 (1.1)
Desensitizer treatment before an ABO-incompatible or HLA-incompatible transplant	—	—	—	2 (1.3)	—	2 (0.7)
Liver transplant	—	—	—	—	2 (3.0)	2 (0.7)
Total A	68 (71.6)	50 (64.9)	3 (100)	99 (62.3)	53 (80.3)	273 (68.3)
B—Not authorized with evidence						
Immunodeficiency replenishment therapy, and blood and blood-forming organs	7 (7.4)	5 (6.5)	0 (0)	24 (15.1)	4 (6.1)	40 (44.0)
Replenishment therapy for secondary antibody deficiency syndrome	4 (4.2)	4 (5.2)	—	16 (10.1)	3 (4.6)	27 (29.7)
Secondary immunodeficiency replacement therapy with severe or recurrent infections, ineffective antimicrobial or specific antibody treatment (PSAF)	3 (3.2)	1 (1.3)	—	8 (5.0)	1 (1.5)	13 (14.3)
Musculoskeletal system and connective tissue	4 (4.3)	6 (7.8)	0 (0)	10 (6.2)	1 (1.5)	21 (23.1)
Dermatomyositis and polymyositis	3 (3.2)	6 (7.8)	—	6 (3.7)	—	15 (16.5)
Systemic lupus erythematosus (SLE)	—	—	—	3 (1.9)	—	3 (3.3)
Stiff person syndrome	1 (1.1)	—	—	1 (0.6)	—	2 (2.2)
Juvenile dermatomyositis with myopathy	—	—	—	—	1 (1.5)	1 (1.1)
Nervous system	8 (8.5)	8 (10.4)	0 (0)	8 (5.1)	2 (3.0)	26 (28.6)
Autoimmune or paraneoplastic encephalitis	—	6 (7.8)	—	4 (2.5)	1 (1.5)	11 (12.1)
Lambert–Eaton myasthemic syndrome	6 (6.4)	1 (1.3)	—	—	—	7 (7.7)
Unspecified myopathy	2 (2.1)	—	—	2 (1.3)	—	4 (4.4)
Severe and refractory epilepsy	—	1 (1.3)	—	—	1 (1.5)	2 (2.2)
Other inflammatory and immune myopathies	—	—	—	2 (1.3)	—	2 (2.2)
Transplantation	1 (1.1)	0 (0)	0 (0)	0 (0)	0 (0)	1 (1.1)
Need for immunization against single bacterial diseases in the context of liver transplantation	1 (1.1)	—	—	—	—	1 (1.1)
Other diseases	1 (1.1)	1 (1.3)	0 (0)	1 (0.6)	0 (0)	3 (3.3)
Replacement therapy in anti-neutrophil cytoplasmic autoantibody (ANCA) vasculitis	1 (1.1)	1 (1.3)	—	1 (0.6)	—	3 (3.3)
Total B	21 (22.1)	20 (26.0)	0 (0)	43 (27.0)	7 (10.1)	91 (22.7)
C—Not authorized without evidence						
Immunodeficiency replenishment therapy, and blood and blood-forming organs	1 (1.1)	0 (0)	0 (0)	1 (0.6)	0 (0)	2 (50.0)
Acquired hemolytic anemia	1 (1.1)	—	—	—	—	1 (25.0)
Unspecified immunodeficiency	—	—	—	1 (0.6)	—	1 (25.0)
Nervous system	1 (1.1)	1 (1.3)	0 (0)	1 (0.6)	0 (0)	2 (50.0)
Demyelinating neuropathy associated with paraproteins (IgM)	1 (1.1)	1 (1.3)	—	—	—	2 (50.0)
Total C	2 (2.1)	1 (1.3)	0 (0)	1 (0.6)	0 (0)	4 (1.0)
UNK—Unknown/not classified						
Total unknown	4 (4.2)	6 (7.8)	0 (0)	16 (10.1)	6 (9.1)	32 (8.0)
Total study cohort (n = 400)	95 (23.8)	77 (19.3)	3 (0.7)	159 (39.7)	66 (16.5)	400 (100)

BUH, Bellvitge University Hospital; GTiPUH, Germans Trias i Pujol University Hospital; SISCAT, Public Health Catalan System; VHUH, Vall d’Hebron University Hospital.

^a^ BUH only has an adult population.

### 3.2 Level of evidence

Stratification by the level of evidence according to SISCAT guidelines showed that most patients, regardless of the age were treated because of an A-level indication (273; 68.3%), followed by B-level (91; 22.7%) and UNK-level (32; 8.0%) indications, and least for a C-level indication (4; 1.0%). Note that almost all pediatric patients (56; 81.2%) were treated under an A-level indication, 7 (10.1%) for a B-level indication, none for a C-level indication, and 6 (8.7%) for UNK-level indications. The proportion of the incident use of an NSIG due to an A-level or C-level evidence indication was higher in the BUH, while in the VHUH, B-level or UNK-level indications were mostly detected (see Table 2). Supplementary Table S4 shows that in VHUH, the percentages of treatment by the level of evidence are quite balanced across sexes. However, in BUH and GTiPUH, we found 15.9%–23.3% fewer women were treated in the A-level category and 11.5%–22.5% more women were treated in the B-level category than men.

When conducting the sensitivity analysis using the classification of levels of evidence of the guideline from the United Kingdom, we perceived a clear shift toward the lower evidence indication categories. Compared to NSIG use based on SISCAT classification, for adults, the A-level usage rate was reduced to one-third. In contrast, the B-level rate increased by 2–3 times and also increased markedly for the UNK-level. C-level indications increased up to 10.4% of adult patients in GTiPUH. In pediatric patients, in GTiPUH, they remained in the A-level. Note that for the VHUH, the proportion of use in the A-level decreased by one-fourth compared to the SISCAT classification, while the B- and UNK-levels significantly increased 5-fold and 2.5-fold, respectively. Additionally, three pediatric patients were now classified into the C-level evidence category (see Supplementary Table S5).

### 3.3 Discontinuation and reasons for discontinuation

From the total population, 37.8% (151) of individuals, 50.7% of pediatrics, and 35.1% of adults discontinued the NSIG treatment. Most pediatric patients who discontinued received NSIGs as replenishment therapy related to a hematopoietic stem-cell transplantation (11; 31.4% of all pediatric discontinuers). For all the hospitals, most discontinuers were treated under the indication of myasthenia gravis, which is classified as an A-level indication by SISCAT guidelines and as a B-level indication by the United Kingdom guidelines: 6 (17.7%), 10 (66.7%), and 9 (13.4%) for the BUH, GTiPUH, and VHUH adult discontinuers, respectively. The majority of patients with authorized or evidence-based indications did not discontinue the therapy. For the other levels of evidence, counts might be insufficient to draw fair inferences (see Supplementary Table S6).

**FIGURE 3:**
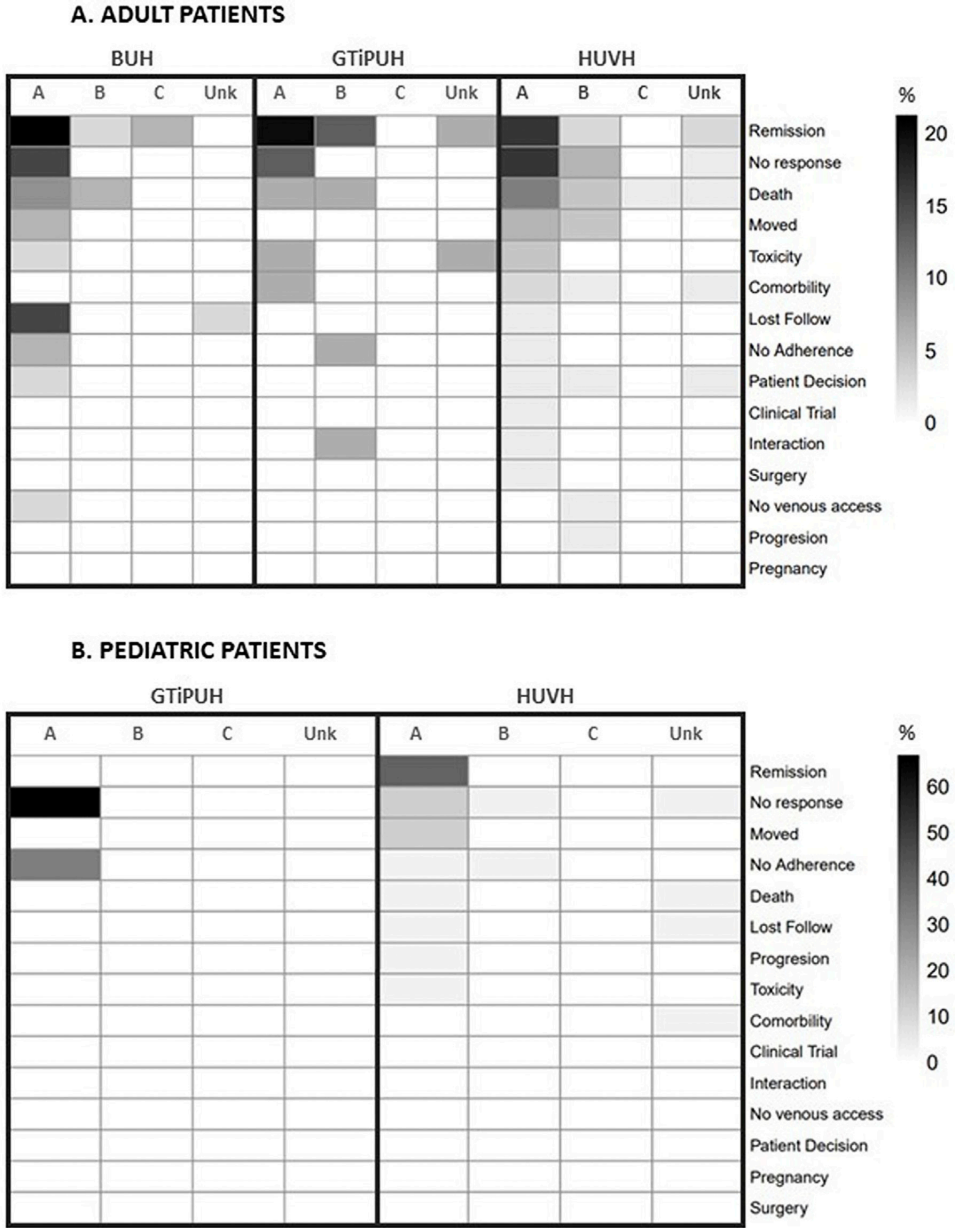
Heatmap showing reasons for discontinuation by hospital, age band, and level of clinical evidence according to SISCAT guidelines. BUH, Bellvitge University Hospital; GTiPUH, Germans Trias i Pujol University Hospital; SISCAT, Public Health Catalan System; VHUH, Vall d'Hebron University Hospital.

The reasons for such discontinuations included remission of the underlying disease or no response to the NSIG therapy, either in adult or pediatric patients. Note that death was the third most common reason for discontinuation, even for A-level and B-level indications of use. There seemed to be no remarkable differences between hospitals (see Figure 3; Supplementary Table S7). In the sensitivity analysis, most discontinuers shifted from the A-level toward the B-level category (see Supplementary Table S8).

### 3.4 Costs

Absolute and patient-visit costs for the main and the sensitivity analyses, and their calculations are provided in Table 3, in euros (€). The included population accumulates a total amount of 2,026,412.8€ in 2.3 years (4.4% for pediatric patients), which means 868,462.6€ per year. Most of the investments are dedicated to A-level indications in the main analysis. Furthermore, it can be seen that costs per patient-visit for the indications with less clinical evidence (C-level and UNK-level) were not negligible at all, especially in GTiPUH and BUH. Nonetheless, spending for UNK-level indications was found in the VHUH as well. In the sensitivity analysis, there is a remarkable shift in spending on A-level indications to levels of lower clinical evidence.

**TABLE 3:** Absolute and patient-visit spending (€) on non-specific immunoglobulins by hospital, age band, and the level of clinical evidence according to SISCAT and United Kingdom guidelines.

		BUH^a^				GTiPUH				VHUH			
Adults (≥18 years of age)													
€ per patient, median [range]^b^		5,062.5 [620.4–31070.7]				5,086.8 [289.5–37727.1]				3,515.6 [455.0–24827.2]			
€ per patient-visit, median [range]^c^		1,656.1 [620.4–8,487.1]				1,627.78 [289.5–9,431.8]				1,272.97 [293.7–12738.9]			
		SISCAT guidelines		United Kingdom guidelines		SISCAT guidelines		United Kingdom guidelines		SISCAT guidelines		United Kingdom guidelines	
		Patient visit^c^	Absolute amount^b^	Patient visit^c^	Absolute amount^b^	Patient visit^c^	Absolute amount^b^	Patient visit^c^	Absolute amount^b^	Patient visit^c^	Absolute amount^b^	Patient visit^c^	Absolute amount^b^
Spending by the level of evidence (€)	A	3,682.2	523,856.7	2,788.4	223,404.5	2,955.6	268,076.6	2,580.3	202,093.9	2,682.8	402,557.5	2,862.1	149,302.8
	B	2,910.3	63,270.3	4,037.7	326,890.5	3,659.3	234,179.4	3,541.5	234,040.1	2,456.0	233,388.3	2,154.8	349,536.6
	C	3,614.9	21,614.5	3,415.9	20,495.4	5,873.1	8,003.3	4,131.2	56,402.4	1,271.7	7,206.3	4,591.0	31,226.8
	Unknown	3,849.6	24,054.8	2,468.6	62,005.9	3,905.1	73,939.3	3,295.6	91,662.1	2,553.6	77,698.9	2,144.0	190,784.7
Overall spending, median €		3,517.2	632,796.2	3,517.2	632,796.2	3,328.2	584,198.6	3,328.2	584,198.6	2,730.4	720,851.0	2,730.4	720,851.0

				BUH^a^					GTiPUH				VHUH			
Pediatrics (<18 years of age)																
€ per patient, median [range]^b^		NA					694.9 [322.2–868.6]					827.2 [132.4–7,815.1]				
€ per patient-visit, median [range]^c^		NA					694.9 [322.2–868.6]					620.4 [82.7–4,136.0]				
		SISCAT guidelines			United Kingdom guidelines		SISCAT guidelines			United Kingdom guidelines		SISCAT guidelines			United Kingdom guidelines	
		Patient visit^c^	Absolute amount^b^		Patient visit^c^	Absolute amount^b^	Patient visit^c^	Absolute amount^b^		Patient visit^c^	Absolute amount^b^	Patient visit^c^		Absolute amount^b^	Patient visit^c^	Absolute amount^b^
Spending by the level of evidence (€)	A	-	-		-	-	628.5	1,885.6		628.5	1,885.6	891.9		72,224.5	1,319.4	34,146.8
	B	-	-		-	-	-	-		-	-	853.9		9,659.20	779.3	38,174.4
	C	-	-		-	-	-	-		-	-	-		-	992.6	2,316.2
	Unknown	-	-		-	-	-	-		-	-	546.0		4,797.8	572.2	12,044.0
Overall spending, median €		0	0		0	0	628.5	1,885.6		628.5	1,885.6	856.4		86,681.4	856.4	86,681.4

BUH, Bellvitge University Hospital; GTiPUH, Germans Trias i Pujol University Hospital; NA, Not applicable; SISCAT, Public Catalan Health System; VHUH, Vall d'Hebron University Hospital.

^a^ BUH only has an adult population.

^b^ To calculate the absolute costs per patient, the costs of all patient visits (NSIG administrations) were summed, and then, the median and range were calculated. These figures are also stratified by the level of evidence and age. Note that the result is time-dependent (the more the visit, the more the expenditure).

^c^ To calculate the costs generated per visit, the cost of each visit for each patient was taken into account, and then, the median and range were calculated. These figures are also stratified by the level of evidence and age. It should be noted that the result does not depend on the length of time the patient was treated.

## 4 Discussion

Our study includes 400 patients from three third-level hospitals in Catalonia, who were mostly treated because of A-level indications. However, non-approved indications are still an issue. Immunodeficiencies, hematopoietic stem cell transplantation, and neurologic diseases were the most frequent diagnoses. The level of evidence classification clearly switched toward lower evidence categories when using United Kingdom clinical guidelines instead. This illustrates that the perspective might vary depending on the reference guideline. The main reasons for discontinuing NSIGs either for pediatric and adult patients were remission of the underlying disease, absence of response, and death. The whole cohort spent 868,462.6€ per year. Most of the investments were in authorized indications, but costs per visit were often higher for unauthorized indications, meaning a significant economic burden on the healthcare system.

Our results on the indications where NSIGs are used are in line with those of other studies (Ruiz-Antorán et al., 2010; Vallejo Rodríguez et al., 1999; Derman et al., 2021b; Albin and Cunningham-Rundles, 2014; Nobile-Orazio et al., 2012; Constantine et al., 2007; So-Osman et al., 2024). The differences we found between the hospitals, such as more neurologic indications in the BUH, more hematologic indications in the VHUH and GTiPUH, and a higher use of SCIGs in the GTiPUH, might be explained by intrinsic characteristics of each, such as the specialties in which one excels or the population in their area of influence. The GTiPUH demonstrated remarkably higher SCIG usage, thanks to an internal protocol supporting SCIG prescriptions and its monitoring, promoting drug safety and savings. Enhancing the use of SCIGs elsewhere could be especially beneficial for patients requiring long-term NSIG replacement, such as immunodeficiencies, as this may allow them reducing the need for hospital visits, increasing compliance, and diminishing the risk of certain adverse drug reactions (ADRs). Even though the prices of SCIGs and IVIGs are similar (see Supplementary Table S2), SCIG use encouragement may reduce costs of healthcare visits (Vultaggio et al., 2015). Protocols and patient training programs should be put in place, and the one from the GTiPUH might be taken as an example.

However, when classifying by the level of evidence, we observed greater heterogenicity. In line with our study, a similar study conducted in 13 tertiary Spanish hospitals over 3 months found that 40% of patients used NSIGs for unauthorized indications. Further investigation into unauthorized use revealed that 39.2% had scientific evidence, while 60.7% lacked evidence (Ruiz-Antorán et al., 2010), which is in contrast to our 71.7% B-level and 28.4% C- and UNK-level indications. However, proportions described in other studies using non-European guidelines ranged from 20% to 62.9% (Constantine et al., 2007; Pendergrast et al., 2005; Chen et al., 2000). These differences might be due to the change in use patterns across time, new evidence, and consequent hospital recommendation trends. Interestingly, sex differences by the level of evidence seem to be explained by the higher prevalence of inflammatory polyneuropathies in men (A-level), particularly CIDP, in agreement with the previous literature (Broers et al., 2019), but targeted studies should be conducted in this regard. Of note, pediatric patients were treated mostly for immunodeficiencies and transplantation-related diseases, which may require long-lasting replacement therapies. Primary immunodeficiencies and immune thrombocytopenic purpura (ITP) affecting children are among the indications with a high level of evidence and prioritized use according to the United Kingdom and SISCAT guidelines (Public Catalan Health System, 2021; Department of Health and Social Care of the Government of United Kingdom, 2011). On the other hand, ITP in children is usually a benign disorder that requires no active management other than careful explanation and counseling. This is because serious bleeding is rare, and about 80% of children with ITP will recover spontaneously within 6–8 weeks. The ability of IVIGs to increase platelet counts in ITP adults is well-supported (Department of Health and Social Care of the Government of United Kingdom, 2011; Tarantino, 2000).

It is important to recognize that a lower level of evidence does not necessarily mean that NSIGs are an inferior option. For example, our B- and C-level indications include secondary immunodeficiencies and myopathies. Secondary immunodeficiencies often arise in patients with hematologic cancers, post-organ transplants, or after certain treatments like chemotherapy. NSIGs are commonly used to reduce infection rates in these cases, especially when standard treatments like antibiotics or preventive vaccines are less effective. For conditions like dermatomyositis and polymyositis, NSIGs are sometimes used when standard treatments (e.g., corticosteroids and immunosuppressive drugs) are inadequate or not well-tolerated. Although NSIGs may have lower levels of evidence, they can be crucial when other treatments fail or are ineffective as they offer broader immune protection, or when severe cases are present and rapid intervention is needed (Touraille and Brosch, 2016; Nobile-Orazio et al., 2012). Although the evidence for NSIGs in these conditions might be weaker than that for other therapies, they often fill a crucial gap, especially in cases where standard treatments fail, are contraindicated, or need to be augmented. Clinicians’ decisions for treatment have to take into account the alternative treatments for a specific indication, even having less definitive evidence when they offer the best chance of improving patient outcomes. This may illustrate the lack of studies for certain conditions.

Even so, the most striking differences regarding evidence classification are explained by the intentions the guidelines on NSIG use are thought for. The majority of guidelines are production–spending-focused when a shortage context happens (Spanish agency for medicines and health products, 2021; Agenzia Italiana del Farmaco, 2022; Agence Nationale de sécurité du médicament et des produits de santé, 2019), while in our study, the clinical evidence on efficacy and effectiveness is core. Note that none of the previous studies focused specifically on pediatric patients; however, some recommendations have been established (Spanish agency for medicines and health products, 2021; Immunodeficiency United Kingdom, 2023; National Blood Authority Australia, 2022; Alberta Ministry of Health Shared Health Manitoba and Saskatchewan Ministry of Health, 2018).

Reasons for discontinuation of NSIGs have been scarcely studied yet only reported as due to adverse effects or indirectly as clinical response (European Medicines Agency, 2016; Wittstock et al., 2003; Guo et al., 2018). However, Ruiz-Antorán et al. (2010) reported 21.4% of 554 patients withdrawing NSIGs due to adverse drug reactions and 9 (1.6%) for lack of response. In comparison, we identified 31 (7.8%) patients from the whole cohort withdrawing due to no response and 7 (1.8%) because of toxicity. Regarding pediatric patients, the most frequent reasons were either remission or non-response, in the A-level indication category. Given that the reasons for discontinuation do not necessarily imply causality, such a lower proportion might be related to recent rationalization policies because of NSIG production shortage as diseases most likely to benefit from the treatment were prioritized. Moreover, non-response might be due to cases where NSIGs were used as an alternative when other alternatives were exhausted, as mentioned previously. Additionally, the rate of use of SCIGs over IVIGs and the use of premedication might have played a role as well (Guo et al., 2018). To the best of our knowledge, the tendency of discontinuation rates for NSIGs and its reasons have not been thoroughly studied, so we did not find further literature reports for comparison.

Regarding spending on NSIGs, we observed relevant investments in medical conditions across all the levels of clinical evidence, which shifted toward lower evidence levels in the sensitivity analysis. The median amount per patient was lower in the VHUH but diminishes when calculating the costs per patient visit. This could be because of a longer person-time follow-up due to more spaced administrations or the use of lower maintenance doses relating to patients’ disease profiles. Such patient-visit costs related to clinical evidence seem consistent with the previous literature (Ruiz-Antorán et al., 2010); however, absolute numbers could not be compared due to different timeframes. Even when considering the costs over the whole study period, ours were higher in the group of authorized indications than in the group of unauthorized indications, unlike the findings obtained by Ruiz-Antorán et al. (2010). It should be taken into consideration that some indications may have been authorized in recent years, hence the relevance of using updated classifications and follow-up over time. Other studies in our setting have evaluated the evolution of NSIG consumption and observed an increase in the patient’s mean costs from 10,930€ in 2010 to 15,595€ in 2021 (Solís-Díez et al., 2022). This contrasts with our calculated costs per patient, which turned out to be half (approximately 5,000€ median €/patient). This difference might be explained by the effect of extreme values skewing the mean upward as we found that spending ranged from a few hundred to approximately 30,000€ per patient. However, this comparison should be taken cautiously, and theirs are costs from estimated annual consumptions, while ours are the actual spending of the centers included. Additionally, as they cover the entire SISCAT, costs are diluted because less severe conditions are treated in smaller hospitals. In contrast, our hospitals serve as reference centers for more severe and less common pathologies, which could imply a higher cost per patient.

Dose reduction strategies for immunoglobulin therapies have shown that reducing doses by 20%–50% can still maintain clinical efficacy in some patients, depending on their condition and severity (Albin and Cunningham-Rundles, 2014). For instance, in the treatment of CIDP, studies suggest that a reduction in IVIG dosing from 2 g/kg to 1 g/kg every 3 weeks can be effective while significantly decreasing overall costs (Derman et al., 2021b). This dose adjustment could result in up to 30%–50% cost savings per patient. Furthermore, when considering alternative therapies, NSIGs have higher priority for the short-term treatment of CIDP than for long-term treatments, particularly during times of shortage (Department of Health and Social Care of the Government of United Kingdom, 2011). Similarly, in primary immunodeficiency diseases, some patients might benefit from reduced doses, with estimated cost savings ranging from 15% to 40%, especially when NSIGs are combined with other alternative therapies (Castle and Robertson, 2019). These examples indicate that substantial cost savings can be achieved while maintaining meaningful clinical benefits by carefully lowering immunoglobulin doses in certain scenarios.

### 4.1 Strengths and limitations

Non-specific immunoglobulins represent a therapeutic option with a high economic cost and limited availability. Not only are associated expenses important but also the potential effectiveness and safety means for the patient. Therefore, one of the strengths of this study is that it provides insights into the clinical practice of immunoglobulins in some of the most representative hospital centers due to the significant influx of treated patients, including specialists in complex diseases. Additionally, we verified the reliability of our data through the review of electronic medical records. Furthermore, replicating our outcomes using another European guideline in our sensitivity analysis helps contextualize the interpretability and enhance comparability against other European countries. Furthermore, NSIG consumption seems to have increased homogenously over time in Europe, but Northern and Western countries have the higher numbers (Marketing Research Bureau, 2023). However, the healthcare system structure should be considered when extrapolating our results to other settings. In decentralized systems, such as Spain, protocols can vary by region, leading to differences in how NSIG therapy is prescribed and funded across different areas, as well as between countries. For this reason, we performed a sensitivity analysis including guidelines from the United Kingdom to increase the comparability and generalizability of our results. Regarding data quality, we performed a structured automated check for data completeness, uniqueness, and consistency. There are scarce studies focusing on the reasons for discontinuation. This is of special concern to pediatric patients, which we handle separately. Moreover, the RPT is a validated source, with 85% correctness and traceable to medical charts (Roig Izquierdo et al., 2020). This makes the registry a reliable source for secondary purposes.

There are some limitations to acknowledge as well. First, the RPT’s accuracy depends on the clinicians who fill it. However, we attempted to address this caveat with the quality-checking process and by accessing medical records. Second, we selected only incident users to remove potential possible memory bias. Third, such a population choice may lead to a reduced ability to evaluate long-term outcomes (Johnson et al., 2012). However, this is offset by the fact that we achieve a lower selection bias in exposure effect estimation than in a prevalent-user design (Luijken et al., 2021). In addition, there is less prevalence bias which normally could lead to a distortion because the association could be the result of the duration of the disease, as opposed to the incident outcome itself (Nour and Plourde, 2019). We encourage follow-up studies combining hospital electronic healthcare information to monitor long-term outcomes and consumption and savings. Finally, the plausibility of the dates of visit was very questionable. In some cases, we were not able to find accurate dates of the follow-up in the medical charts matching the RPT. To tackle this, we amended inexact dates in the RPT with those in the medical charts and calculated the cost estimates using the number of visits instead of date variables, given that they relate to a dose administration. Furthermore, costs per patient visit may ease the comparison with other studies because of not being influenced by the sample size and time. Although we report costs granularly, fair comparisons with the existing literature were difficult as cost calculations are not reported in detail sometimes. Additionally, the prices per gram we used are those agreed between the hospital and the manufacturer, which might limit generalization. Currently, regular meetings are in place for close case-by-case monitoring.

In conclusion, immunoglobulins represent a therapeutic option with high economic costs and limited availability. They are mainly used for approved indications like nervous system and hematologic diseases in clinical practice; however, there is still a proportion used in non-approved indications. This could still represent a significant economic burden on the healthcare system. Hopefully, our efforts could, in turn, lead to the optimization of the use of immunoglobulins with a particular focus on the unique needs of the pediatric population and those at risk for discontinuation with alternative therapeutic options. Furthermore, our results show that the perspective might vary depending on the guideline used as reference, so the harmonization of the clinical evidence assessment at a European level could lead to a deeper understanding for regulatory entities and guideline makers to optimize the use of immunoglobulins.

## Data Availability

The data analyzed in this study are subject to the following licenses/restrictions: the datasets presented in this article are not readily available because this study used anonymized patient data from electronic healthcare databases. These data remain local within each center as local data protection regulations for individual data privacy protection apply. Requests to access these datasets should be directed to Mònica Sabaté, monica.sabate@vallhebron.cat.

## References

[B1] Agence Nationale de sécurité du médicament et des produits de santé (ANSM) (2019). Use of multivalent human immunoglobulins (Ig) in a context of supply tension: update on the actions implemented. Available at: https://ansm.sante.fr/actualites/utilisation-des-immunoglobulines-humaines-polyvalentes-ig-dans-un-contexte-de-tensions-dapprovisionnement-point-sur-les-actions-mises-en-oeuvre (Accessed March 24, 2024).

[B2] Agenzia Italiana del Farmaco (AIFA) (2022). Guideline on the use of human immunoglobulins in case of shortages. Available at: https://www.aifa.gov.it/documents/20142/847339/Guidelines_on_the_use_of_human_immunoglobulins_in_case_of_shortages.pdf (Accessed April 2, 2024).

[B3] Alberta Ministry of Health Shared Health Manitoba and Saskatchewan Ministry of Health (2018). “Prairie collaborative immune globulin utilization management framework project,” in Criteria for the clinical use of immune globulin. Available at: https://www.ihe.ca/download/criteria_for_the_clinical_use_of_immune_globulin_first_edition.pdf (Accessed March 24, 2024).

[B4] Albin S. Cunningham-Rundles C. (2014). An update on the use of immunoglobulin for the treatment of immunodeficiency disorders. Immunotherapy 6, 1113–1126. 10.2217/imt.14.67 25428649 PMC4324501

[B5] AminJafari A. Ghasemi S. (2020). The possible of immunotherapy for COVID-19: a systematic review. Int. Immunopharmacol. 83, 106455. 10.1016/j.intimp.2020.106455 32272396 PMC7128194

[B6] Angelotti F. Capecchi R. Giannini D. Mazzarella O. Rocchi V. Migliorini P. (2020). Long-term efficacy, safety, and tolerability of recombinant human hyaluronidase-facilitated subcutaneous infusion of immunoglobulin (Ig) (fSCIG; HyQvia(®)) in immunodeficiency diseases: real-life data from a monocentric experience. Clin. Exp. Med. 20, 387–392. 10.1007/s10238-020-00633-4 32385734

[B7] Bellvitge Hospital Universitari (2024). Available at: https://bellvitgehospital.cat/es/quienes-somos/historia (Accessed April 5, 2024).

[B8] Broers M. C. Bunschoten C. Nieboer D. Lingsma H. F. Jacobs B. C. (2019). Incidence and prevalence of chronic inflammatory demyelinating polyradiculoneuropathy: a systematic review and meta-analysis. Neuroepidemiology 52, 161–172. 10.1159/000494291 30669140 PMC6518865

[B9] Canadian blood service (2021). Plasma and the blood system supply chain. Available at: https://www.blood.ca/en/about-us/media/newsroom/plasma-and-blood-system-supply-chain (Accessed March 23, 2024).

[B10] Castle D. Robertson N. P. (2019). Alternatives to intravenous immunoglobulin treatment in chronic inflammatory demyelinating polyradiculoneuropathy. J. Neurol. 266 (9), 2338–2340. 10.1007/s00415-019-09485-9 31372734 PMC6687691

[B11] Chen C. Danekas L. H. Ratko T. A. Vlasses P. H. Matuszewski K. A. (2000). A multicenter drug use surveillance of intravenous immunoglobulin utilization in US academic health centers. Ann. Pharmacother. 34, 295–299. 10.1345/aph.19252 10917372

[B12] Condino-Neto A. Costa-Carvalho B. T. Grumach A. S. King A. Bezrodnik L. Oleastro M. (2014). Guidelines for the use of human immunoglobulin therapy in patients with primary immunodeficiencies in Latin America. Allergol. Immunopathol. Madr. 42, 245–260. 10.1016/j.aller.2012.09.006 23333411

[B13] Constantine M. M. Thomas W. Whitman L. Kahwash E. Dolan S. Smith S. (2007). Intravenous immunoglobulin utilization in the Canadian atlantic provinces: a report of the atlantic collaborative intravenous immune globulin utilization working group. Transfusion 47, 2072–2080. 10.1111/j.1537-2995.2007.01400.x 17958537

[B14] Department of Health and Social Care of the Government of United Kingdom (2011). Clinical guidelines for immunoglobulin use. second ed update. Available at: https://www.gov.uk/government/publications/clinical-guidelines-for-immunoglobulin-use-second-edition-update (Accessed April 2, 2024).

[B15] Derman B. A. Schlei Z. Parsad S. Mullane K. Knoebel R. W. (2021a). Changes in intravenous immunoglobulin usage for hypogammaglobulinemia after implementation of a stewardship program. JCO Oncol. Pract. 17, e445–e453. 10.1200/op.20.00312 32822257 PMC8257910

[B16] Derman B. A. Schlei Z. Parsad S. Mullane K. Knoebel R. W. (2021b). Changes in intravenous immunoglobulin usage for hypogammaglobulinemia after implementation of a stewardship program. JCO Oncol. Pract. 17, e445–e453. 10.1200/op.20.00312 32822257 PMC8257910

[B17] European Medicines Agency (EMA) (2016). Pharmacovigilance Risk Assessment Committee (PRAC) report. Reflection paper on collecting and reporting information on off-label use in pharmacovigilance. Available at: https://www.ema.europa.eu/en/documents/regulatory-procedural-guideline/reflection-paper-collecting-reporting-information-label-use-pharmacovigilance_en.pdf (Accessed March 23, 2024).

[B18] European Parliament (2020). Report on the shortage of medicines – how to address an emerging problem. Available at: https://www.europarl.europa.eu/doceo/document/A-9-2020-0142_EN.html ([Accessed February 28, 2024).

[B19] Farrugia A. Poulis P. (2001). Intravenous immunoglobulin: regulatory perspectives on use and supply. Transfus. Med. 11 (1), 63–74. 10.1046/j.1365-3148.2001.00288.x 11299022

[B20] Guo Y. Tian X. Wang X. Xiao Z. (2018). Adverse effects of immunoglobulin therapy. Front. Immunol. 9, 1299. 10.3389/fimmu.2018.01299 29951056 PMC6008653

[B21] Hospital Universitari Germans Trias i Pujol (2024). Available at: https://www.hospitalgermanstrias.cat/(Accessed April 4, 2024).

[B22] Immunodeficiency UK (United Kingdom) (2023). Immunoglobulin replacement therapy. Available at: http://www.immunodeficiencyuk.org/whatarepids/treatment/immunoglobulinreplacementtherapy/igavailabiity (Accessed February 17, 2024).

[B23] Johnson E. S. Bartman B. A. Briesacher B. A. Fleming N. S. Gerhard T. Kornegay C. J. (2012). The incident user design in comparative effectiveness research. Pharmacoepidemiol. Drug Saf 22 (1), 1–6. 10.1002/pds.3334 23023988

[B24] Luijken K. Spekreijse J. J. van Smeden M. Gardarsdottir H. Groenwold R. H. H. (2021). New‐user and prevalent‐user designs and the definition of study time origin in pharmacoepidemiology: a review of reporting practices. Pharmacoepidemiol Drug Saf. 30, 960–974. 10.1002/pds.5258 33899305 PMC8252086

[B25] Marketing Research Bureau (2023). EU NSIG therapy usage and funding. Available at: https://marketingresearchbureau.com/wp-content/uploads/2023/05/MRB_EU_SOHO_Figures1.pdf (Accessed October 11, 2024).

[B26] National Blood Authority Australia (2022). Bloodstar. Ig governance. Criteria Clin. Use Immunoglobulin Aust. Available at: https://www.criteria.blood.gov.au/CheckEligibility (Accessed November 9, 2023).

[B27] N’kaoua E. Attarian S. Delmont E. Campana-Salort E. Verschueren A. Grapperon A. M. (2022). Immunoglobulin shortage: practice modifications and clinical outcomes in a reference centre. Rev. Neurol. 178, 616–623. 10.1016/j.neurol.2021.10.004 34872746

[B28] Nobile-Orazio E. Cocito D. Jann S. Uncini A. Beghi E. Messina P. (2012). Intravenous immunoglobulin versus intravenous methylprednisolone for chronic inflammatory demyelinating polyradiculoneuropathy: a randomised controlled trial. Lancet Neurol. 11, 493–502. 10.1016/s1474-4422(12)70093-5 22578914

[B29] Nour S. Plourde G. (2019). “Pharmacoepidemiology in the prevention of adverse drug reactions,” in Pharmacoepidemiology and pharmacovigilance. Editors Nour, S. Plourde G. (Academic Press), 25–65.

[B30] Pendergrast J. M. Sher G. D. Callum J. L. (2005). Changes in intravenous immunoglobulin prescribing patterns during a period of severe product shortages, 1995–2000. Vox Sang. 89, 150–160. 10.1111/j.1423-0410.2005.00670.x 16146507

[B31] Public Catalan Health System (SISCAT) (2021). Utilització i adequació clínica de les immunoglobulines inespecífiques a l’àmbit del SISCAT – 2n actualització. [Accessed April 2, 2024].

[B32] Roig Izquierdo M. Prat Casanovas M. A. Gorgas Torner M. Q. Pontes García C. (2020). Registry of patients and treatments of hospital medicines in Spain: 10 years of clinical data. Med. Clin. Engl. Ed. 154, 185–191. 10.1016/j.medcli.2019.09.009 31759696

[B33] RStudio Team (2020). RStudio version 4.1.2. Integrated Development for R. Vienna, Austria: R Foundation for Statistical Computing. Available at: https://www.R-project.org/(Accessed April 2, 2024).

[B46] Ruiz-Antorán B. Agustí Escasany A. Vallano Ferraz A. Danés Carreras I. Riba N. Mateu Escudero S. (2010). Use of non-specific intravenous human immunoglobulins in Spanish hospitals; need for a hospital protocol. Eur. J. Clin. Pharmacol. 66 (6), 633–641. 10.1007/s00228-010-0800-y 20204337

[B34] Solís-Díez G. Turu-Pedrola M. Roig-Izquierdo M. Zara C. Vallano A. Pontes C. (2022). Dealing with immunoglobulin shortages: a rationalization plan from evidence-based and data collection. Front. Public Health 10, 893770. 10.3389/fpubh.2022.893770 35664094 PMC9160570

[B35] So-Osman C. Delaney M. Fung M. Lu W. Murphy M. Sasongko P. L. (2024). A global analysis of the use of immunoglobulin, shortages in supply, and mitigating measures: a survey of hospital providers (a BEST Collaborative study). Transfusion 64, 775–783. 10.1111/trf.17801 38516758

[B36] Spanish agency for medicines and health products (AEMPS) (2021). Semi-annual report of medicines shortages. Available at: https://www.aemps.gob.es/medicamentosUsoHumano/problemasSuministro/informes-semestrales/docs/primer-informe-semestral-2021.pdf?x99230 (Accessed: November 24, 2023).

[B37] Stanworth S. J. New H. V. Apelseth T. O. Brunskill S. Cardigan R. Doree C. (2020). Effects of the COVID-19 pandemic on supply and use of blood for transfusion. Lancet Haematol. 7 (9), e756–e764. 10.1016/S2352-3026(20)30186-1 32628911 PMC7333996

[B38] Tarantino M. D. (2000). Treatment options for chronic immune (idiopathic) thrombocytopenia purpura in children. Semin. Hematol. 37, 35–41. 10.1016/s0037-1963(00)90117-3 10676923

[B39] Toumi M. Urbinati D. (2015). An EU-wide overview of the market of blood, blood components plasma derivatives focusing on their availability for patients. Creative Ceutical Rep. Revis. by Comm. Incl. Stakeholders' Comments. Available at: https://health.ec.europa.eu/system/files/2016-11/20150408_cc_report_en_0.pdf ([Accessed April 9, 2024).

[B40] Touraille G. Brosch S. (2016). Collecting and reporting information on off-label use. Available at: https://www.ema.europa.eu/en/documents/presentation/presentation-collecting-reporting-information-label-use-gilles-touraille-sabine-brosch_en.pdf (Accessed November 23, 2023).

[B41] Vall D’Hebron Barcelona Hospital Campus (2024). Available at: https://www.vallhebron.com/(Accessed April 4, 2024).

[B42] Vallejo Rodríguez I. Socias Manzano M. S. López Arranz C. Mangues Bafalluy I. Marín Pozo J. F. Sacristán de Lama M. P. (1999). Inmunoglobulinas de administración intravenosa. Actualización de sus indicaciones. Farm Hosp. 23 (5), 271–288.

[B43] Vultaggio A. Azzari C. Milito C. Finocchi A. Toppino C. Spadaro G. (2015). Subcutaneous immunoglobulin replacement therapy in patients with primary immunodeficiency in routine clinical practice: the VISPO prospective multicenter study. Clin. Drug Investig. 35, 179–185. 10.1007/s40261-015-0270-1 PMC4335091 25672929

[B44] Wittstock M. Benecke R. Zettl U. K. (2003). Therapy with intravenous immunoglobulins: complications and side-effects. Eur. Neurol. 50, 172–175. 10.1159/000073059 14530624

[B45] Wörner N. Rodrigo-García R. Antón A. Castellarnau E. Delgado I. Vazquez È. (2021). Enterovirus-A71 rhombencephalitis outbreak in Catalonia: characteristics, management and outcome. Pediatr. Infect. Dis. J. 40 (6), 628–633. 10.1097/inf.0000000000003114 34097655 PMC8189429

