# Supplementary material for: Use of non-specific immunoglobulins in Catalonia in three third-level hospitals: a descriptive analysis of a hospital-prescribed medication registry
Source: Front Pharmacol. 2024 Dec 16;15:1420682. doi: 10.3389/fphar.2024.1420682 (PMC11682906; doi:10.3389/fphar.2024.1420682)
Supplement: Supplementary file 1 [file Table1.DOCX]

| *Table S1. Classification of indications by level of evidence according to SISCAT guidelines and United Kingdom guidelines.* | | | | | | |
| --- | --- | --- | --- | --- | --- | --- |
| **ICD10 Diagnostic group** | **ICD10 Diagnostic subgroup** | **ICD10 Diagnotic code** | **Indication** | **Description of the diagnosis** | **Level of evidence by SISCAT guidelines** | **Level of evidence by United Kingdom guidelines** |
| Certain infectious and parasitic diseases | Nocardiosis | A430 | Pulmonary nocardiosis | Pulmonary nocardiosis | Unknown | Unknown |
| Infectious and parasitic diseases | Viral infection of unspecified site | B349 | Viral infection, unspecified | Viral infection, unspecified | Unknown | Unknown |
| Neoplasms | Hodgkin lymphoma | C8112 | Nodular sclerosis Hodgkin lymphoma, intrathoracic lymph nodes | Nodular sclerosis Hodgkin lymphoma, intrathoracic lymph nodes | Unknown | Unknown |
|  | Other and unspecified types of non-Hodgkin lymphoma | C8580 | Other specified types of non-Hodgkin lymphoma, unspecified site | Other specified types of non-Hodgkin lymphoma, unspecified site | Unknown | Unknown |
|  | Multiple myeloma and malignant plasma cell neoplasms | C90 | Replenishment therapy multiple myeloma with severe secondary hypogammaglobulinemia and recurrent infections | Multiple myeloma and malignant plasma cell neoplasms | A | B |
|  | Lymphoid leukaemia | C91 | Replenishment therapy chronic lymphatic leukemia with severe secondary hypogammaglobulinemia and recurrent infections | Lymphoid leukemia | A | B |
|  | Lymphoid leukaemia | C9102 | Acute lymphoblastic leukemia, in relapse | Acute lymphoblastic leukemia, in relapse | Unknown | Unknown |
| Diseases of the blood and blood-forming organs and certain disorders involving the immune mechanism | Acquired haemolytic anaemia | D599 | Acquired hemolytic anemia, unspecified | Acquired hemolytic anemia, unspecified | C | C |
|  | Other coagulation defects | D68312 | Antiphospholipid antibodies with hemorrhagic disorder | Antiphospholipid antibody with hemorrhagic disorder | Unknown | Unknown |
|  | Purpura and other haemorrhagic conditions | D69 | Immunomodulation - Immune thrombocytopenic purpura (ITP) with impaired antibody production | Purpura and other hemorrhagic conditions | A | A |
|  | Purpura and other haemorrhagic conditions | D69 | Immunomodulation - Primary immune thrombocytopenia with risk of bleeding | Purpura and other hemorrhagic conditions | A | Unknown |
|  | Purpura and other haemorrhagic conditions | D693 | Idiopathic thrombocytopenic purpura | Idiopathic thrombocytopenic purpura | A | A |
|  | Immunodeficiency with predominantly antibody defects | D80 | Replenishment therapy in SIP - IgG subclass deficiency with recurrent infections | Immunodeficiency with predominantly antibody defects | A | A |
|  | Immunodeficiency with predominantly antibody defects | D80 | Replenishment therapy in SIP - Congenital hypogammaglobulinemias | Immunodeficiency with predominantly antibody defects | A | A |
|  | Immunodeficiency with predominantly antibody defects | D801 | Nonfamilial hypogammaglobulinemia | Nonfamilial hypogammaglobulinemia | A | Unknown |
|  | Combined immunodeficiencies | D81 | Replenishment therapy in SIP - Combined severe immunodeficiency | Combined immunodeficiencies | A | A |
|  | Immunodeficiency associated with other major defects | D82 | Replenishment therapy for secondary antibody deficiency syndrome | Immunodeficiency associated with other major defects | B | B |
|  | Immunodeficiency associated with other major defects | D82 | Secondary immunodeficiency replacement therapy with severe or recurrent infections, ineffective antimicrobial treatment and proven failure of specific antibodies (PSAF) | Immunodeficiency associated with other major defects | B | B |
|  | Common variable immunodeficiency | D83 | Replacement therapy in SIP - Common variable immunodeficiency | Common variable immunodeficiency | A | A |
|  | Other immunodeficiencies | D84 | Replacement therapy for primary antibody immunodeficiency syndrome (SIP) - non-specific | Other immunodeficiencies | A | Unknown |
|  | Other immunodeficiencies | D849 | Immunodeficiency, unspecified | Immunodeficiency, unspecified | C | Unknown |
|  | Other disorders involving the immune mechanism, not elsewhere classified | D891 | Cryoglobulinemia | Cryoglobulinemia | Unknown | Unknown |
|  | Other disorders involving the immune mechanism, not elsewhere classified | D8982 | Autoimmune lymphoproliferative syndrome [SLPA] | Autoimmune lymphoproliferative syndrome [ALPS] | Unknown | Unknown |
| Diseases of the nervous system | Encephalitis, myelitis and encephalomyelitis | G04 | Autoimmune or paraneoplastic encephalitis | Encephalitis, myelitis and encephalomyelitis | B | C |
|  | Other extrapyramidal and movement disorders | G25 | Stiff person syndrome | Other extrapyramidal and movement disorders | B | B |
|  | Other acute disseminated demyelination | G36 | Other acute disseminated demyelination | Other acute disseminated demyelination | Unknown | Unknown |
|  | Epilepsy | G40 | Severe and refractory epilepsy | Epilepsy | B | C |
|  | Inflammatory polyneuropathy | G61 | Demyelinating neuropathy associated with paraproteins (IgM) | Inflammatory polyneuropathy | C | A |
|  | Inflammatory polyneuropathy | G61 | Immunomodulation - Multifocal motor neuropathy | Inflammatory polyneuropathy | A | B |
|  | Inflammatory polyneuropathy | G61 | Immunomodulation - Chronic inflammatory demyelinating polyneuropathy | Inflammatory polyneuropathy | A | A |
|  | Inflammatory polyneuropathy | G61 | Immunomodulation - Guillain Barré syndrome | Inflammatory polyneuropathy | A | A |
|  | Inflammatory polyneuropathy | G6189 | Other inflammatory polyneuropathies | Other inflammatory polyneuropathies | A | Unknown |
|  | Other polyneuropathies | G629 | Unspecified polyneuropathy | Polyneuropathy, unspecified | Unknown | Unknown |
|  | Myasthenia gravis and other myoneural disorders | G70 | Myasthenia gravis | Myasthenia gravis and other myoneural disorders | A | B |
|  | Myasthenia gravis and other myoneural disorders | G70 | Lambert-Eaton myasthemic syndrome | Myasthenia gravis and other myoneural disorders | B | B |
|  | Myasthenia gravis and other myoneural disorders | G7000 | Myasthenia gravis without (acute) exacerbation | Myasthenia gravis without (acute) exacerbation | A | B |
|  | Primary disorders of muscles | G712 | Congenital myopathies | Congenital myopathies | Unknown | Unknown |
|  | Other myopathies | G7249 | Other inflammatory and immune myopathies, not elsewhere classified | Other inflammatory and immune myopathies, not elsewhere classified | B | Unknown |
|  | Other myopathies | G729 | Myopathy, unspecified | Myopathy, unspecified | B | Unknown |
| Diseases of the circulatory system | Cerebral infarction | I6350 | Cerebral infarction due to unspecified occlusion or stenosis of unspecified cerebral artery | Cerebral infarction due to unspecified occlusion or stenosis of unspecified cerebral artery | Unknown | Unknown |
|  | Cerebral infarction | I639 | Cerebral infarction, unspecified | Cerebral infarction, unspecified | Unknown | Unknown |
|  | Other cerebrovascular diseases | I677 | Cerebral arteritis, not elsewhere classified | Cerebral arteritis, not elsewhere classified | Unknown | Unknown |
|  | Other disorders of arteries and arterioles | I77 | Replacement therapy in ANCA vasculitis | Other disorders of arteries and arterioles | B | C |
| Diseases of the skin and subcutaneous tissue | Impetigo | L0100 | Impetigo, unspecified | Impetigo, unspecified | Unknown | Unknown |
|  | Pemphigoid | L120 | Bullous pemphigoid | Bullous pemphigoid | Unknown | Unknown |
|  | Vasculitis limited to skin, not elsewhere classified | L959 | Vasculitis limited to the skin, unspecified | Vasculitis limited to the skin, unspecified | Unknown | Unknown |
| Diseases of the musculoskeletal system and connective tissue | Other autoimmflamatory syndromes | M048 | Other autoinflammatory syndromes | Other autoinflammatory syndromes | Unknown | Unknown |
|  | Systemic lupus erythematosus | M32 | Systemic lupus erythematosus (SLE) | Systemic lupus erythematosus (SLE) | B | B |
|  | Dermatopolymyositis | M3302 | Juvenile dermatomyositis with myopathy | Juvenile dermatomyositis with myopathy | B | B |
|  | Dermatopolymyositis | M3322 | Polymyositis with myopathy | Polymyositis with myopathy | B | Unknown |
|  | Dermatopolymyositis | M3390 | Dermatopolymyositis, unspecified, organ involvement unspecified | Dermatopolymyositis, unspecified, organ involvement unspecified | B | B |
|  | Dermatopolymyositis | M3391 | Dermatopolymyositis, unspecified with respiratory involvement | Dermatopolymyositis, unspecified with respiratory involvement | B | B |
|  | Systemic sclerosis | M340 | Progressive systemic sclerosis | Progressive systemic sclerosis | Unknown | Unknown |
|  | Systemic sclerosis | M349 | Systemic sclerosis, unspecified | Systemic sclerosis, unspecified | Unknown | Unknown |
|  | Other systemic involvement of connective tissue | M3500 | Sjögren syndrome, unspecified | Sjögren syndrome, unspecified | Unknown | Unknown |
|  | Systemic disorders of connective tissue in diseases classified elsewhere | M360 | Dermato(poly)myositis in neoplastic disease | Dermato(poly)myositis in neoplastic disease | B | B |
|  | Ankylosing spondylitis | M459 | Ankylosing spondylitis of unspecified sites in spine | Ankylosing spondylitis of unspecified sites in spine | Unknown | Unknown |
|  | Myositis | M609 | Myositis, unspecified | Myositis, unspecified | Unknown | Unknown |
| Diseases of the genitourinary system | Other renal tubulo-interstitial diseases | N158 | Other specified renal tubulo-interstitial diseases | Other specified renal tubulo-interstitial diseases | Unknown | Unknown |
|  | Chronic kidney disease | N186 | End stage renal disease | End stage renal disease | Unknown | Unknown |
| Pregnancy, childbirth and the puerperium | Supervision of pregnancy with other poor reproductive or obstetric history | O09299 | Supervision of pregnancy with other poor reproductive or obstetric history, unspecified trimester | Supervision of pregnancy with other poor reproductive or obstetric history, unspecified trimester | Unknown | Unknown |
|  | Single spontaneous delivery | O80 | Single spontaneous delivery | Single spontaneous delivery | Unknown | Unknown |
| Symptoms, signs and abnormal clinical and laboratory findings | Abnormalities of breathing | R0600 | Dyspnea, unspecified | Dyspnea, unspecified | Unknown | Unknown |
|  | Ascites | R180 | Malignant ascites | Malignant ascites | Unknown | Unknown |
|  | Unknown and unspecified causes of morbidity | R69 | Unknown and unspecified causes of morbidity | Unknown and unspecified causes of morbidity | Unknown | Unknown |
| Injury, poisoning and certain other consequences of external causes | Injury of other and unspecified intrathoracic organs | S27301S | Unspecified injury of lung, unilateral | Unspecified injury of lung, unilateral | Unknown | Unknown |
|  | Failure and rejection of transplanted organs and tissues | T8611 | Kidney transplant rejection | Kidney transplant rejection | A | Unknown |
|  | Failure and rejection of transplanted organs and tissues | T8691 | Antibody-mediated rejection treatment in solid organ transplant | Unspecified transplanted organ and tissue rejection | A | B |
| Factors influencing health status and contact with health services | Need for immunization against single bacterial diseases | Z23 | Need for immunization against single bacterial diseases in the context of liver transplantation | Need for immunization against single bacterial diseases | B | Unknown |
|  | Family history of certain disabilities and chronic diseases leading to disablement | Z8279 | Family history of other congenital malformations, deformations and chromosomal abnormalities | Family history of other congenital malformations, deformations and chromosomal abnormalities | Unknown | Unknown |
|  | Transplanted organ and tissue status | Z94 | Desensitizer treatment before a high-risk immunological solid organ transplant | Transplanted organ and tissue status | A | B |
|  | Transplanted organ and tissue status | Z94 | Desensitizer treatment before an ABO-incompatible or HLA-incompatible transplant | Transplanted organ and tissue status | A | B |
|  | Transplanted organ and tissue status | Z940 | Kidney transplant status | Kidney transplant status | A | Unknown |
|  | Transplanted organ and tissue status | Z944 | Liver transplant status | Liver transplant status | A | Unknown |
|  | Transplanted organ and tissue status | Z9481 | Bone marrow transplant status | Bone marrow transplant status | A | B |
|  | Transplanted organ and tissue status | Z9484 | Replenishment therapy before and after allogeneic hematopoietic stem cell transplantation with hypogammaglobulinemia | Stem cells transplant status | A | B |
| *ICD10: International Classification of Diseases, Tenth Revision; SISCAT: Public Catalan Health System* | | | | | |  |
