# Supplementary material for: Use of non-specific immunoglobulins in Catalonia in three third-level hospitals: a descriptive analysis of a hospital-prescribed medication registry
Source: Front Pharmacol. 2024 Dec 16;15:1420682. doi: 10.3389/fphar.2024.1420682 (PMC11682906; doi:10.3389/fphar.2024.1420682)
Supplement: Supplementary file 3 [file Table7.docx]

| *Table S7. Reasons for discontinuation of people withdrawing therapy with non-specific immunoglobulins, by level of evidence based on SISCAT's guidelines, ageband and hospital. Number of discontinuers per level of evidence, ageband and hospital are used as denominator. Empty cells mean no cases were identified.* | | | | | | | | | | | | | |
| --- | --- | --- | --- | --- | --- | --- | --- | --- | --- | --- | --- | --- | --- |
|  | **BUH (n=34)** | | | | **GTiPUH (n=18)** | | | | **VHUH (n=99)** | | | |  |
| **Reasons for discontinuation** | **A** | **B** | **C** | **Unknown** | **A** | **B** | **C** | **Unknown** | **A** | **B** | **C** | **Unknown** |  |
| ***Adults (≥18 years of age)*** | | | | | | | | | | | | | **TOTAL ADULTS, n(%)** |
| Remission | 9 (32.1) | 1 (33.4) | 2 (100) |  | 3 (37.5) | 2 (40.0) |  | 1 (50.0) | 11 (25.0) | 2 (12.5) |  | 2 (33.4) | **33 (28.5)** |
| No response | 5 (17.9) |  |  |  | 2 (25.0) |  |  |  | 11 (25.0) | 4 (25.0) |  | 1 (16.7) | **23 (19.8)** |
| Death | 2 (7.1) | 2 (66.6) |  |  | 1 (12.5) | 1 (20.0) |  |  | 7 (15.9) | 3 (18.7) | 1 (100) | 1 (16.7) | **18 (15.5)** |
| Moved | 2 (7.1) |  |  |  |  |  |  |  | 4 (9.1) | 3 (18.7) |  |  | **9 (7.8)** |
| Lost Follow | 5 (17.9) |  |  | 1 (100) |  |  |  |  | 1 (2.3) |  |  |  | **7 (6.0)** |
| Toxicity | 1 (3.6) |  |  |  | 1 (12.5) |  |  | 1 (50.0) | 3 (6.8) |  |  |  | **6 (5.2)** |
| Comorbility |  |  |  |  | 1 (12.5) |  |  |  | 2 (4.5) | 1 (6.3) |  | 1 (16.7) | **5 (4.3)** |
| No adherence | 2 (7.1) |  |  |  |  | 1 (20.0) |  |  | 1 (2.3) |  |  |  | **4 (3.5)** |
| Patient decision | 1 (3.6) |  |  |  |  |  |  |  | 1 (2.3) | 1 (6.3) |  | 1 (16.7) | **4 (3.5)** |
| No venous access | 1 (3.6) |  |  |  |  |  |  |  |  | 1 (6.3) |  |  | **2 (1.7)** |
| Interaction |  |  |  |  |  | 1 (20.0) |  |  | 1 (2.3) |  |  |  | **2 (1.7)** |
| Progresion |  |  |  |  |  |  |  |  |  | 1 (6.3) |  |  | **1 (0.9)** |
| Surgery |  |  |  |  |  |  |  |  | 1 (2.3) |  |  |  | **1 (0.9)** |
| Clinical trial |  |  |  |  |  |  |  |  | 1 (2.3) |  |  |  | **1 (0.9)** |
| Pregnancy |  |  |  |  |  |  |  |  |  |  |  |  | **0 (0)** |
| **TOTAL PER LoE** | **28 (82.4)** | **3 (8.8)** | **2 (5.9)** | **1 (2.9)** | **8 (53.3)** | **5 (33.3)** | **0 (0)** | **2 (13.4)** | **44 (65.7)** | **16 (23.8)** | **1 (1.5)** | **6 (9.0)** | **116 (100)** |
| **TOTAL ADULTS PER HOSPITAL** | **34 (29.3)** | | | | **15 (12.9)** | | | | **67 (57.8)** | | | |  |
|  | | | | | | | | | | | | | |
| ***Pediatrics (<18 years of age)*** | | | | | | | | | | | | | **TOTAL PEDIATRICS, n(%)** |
| Remission |  |  |  |  |  |  |  |  | 13 (50.0) |  |  |  | **13 (37.1)** |
| No response |  |  |  |  | 2 (66.6) |  |  |  | 4 (15.4) | 1 (50.0) |  | 1 (25.0) | **8 (22.9)** |
| Moved |  |  |  |  |  |  |  |  | 4 (15.4) |  |  |  | **4 (11.4)** |
| No adherence |  |  |  |  | 1 (33.4) |  |  |  | 1 (3.8) | 1 (50.0) |  |  | **3 (8.6)** |
| Death |  |  |  |  |  |  |  |  | 1 (3.8) |  |  | 1 (25.0) | **2 (5.7)** |
| Lost Follow |  |  |  |  |  |  |  |  | 1 (3.8) |  |  | 1 (25.0) | **2 (5.7)** |
| Comorbility |  |  |  |  |  |  |  |  |  |  |  | 1 (25.0) | **1 (2.9)** |
| Toxicity |  |  |  |  |  |  |  |  | 1 (3.8) |  |  |  | **1 (2.9)** |
| Progresion |  |  |  |  |  |  |  |  | 1 (3.8) |  |  |  | **1 (2.9)** |
| Other reasons* |  |  |  |  |  |  |  |  |  |  |  |  | **0 (0)** |
| **TOTAL PER LoE** | **0 (0)** | **0 (0)** | **0 (0)** | **0 (0)** | **3 (100)** | **0 (0)** | **0 (0)** | **0 (0)** | **26 (81.3)** | **2 (6.3)** | **0 (0)** | **4 (12.5)** | **35 (100)** |
| **TOTAL PEDIATRICS PER HOSPITAL** | **0 (0)** | | | | **3 (8.6)** | | | | **32 (91.4)** | | | |  |
| **Other reasons include: patient decision, surgery, clinical trial, pregnancy, no venous access or interaction.* | | | | | | | | | | | | |  |
| *BUH: Bellvitge University Hospital; GTiPUH: Germans Trias i Pujol University Hospital; VHUH: Vall d'Hebron University Hospital* | | | | | | | | | | | | | |
