# Supplementary material for: Use of non-specific immunoglobulins in Catalonia in three third-level hospitals: a descriptive analysis of a hospital-prescribed medication registry
Source: Front Pharmacol. 2024 Dec 16;15:1420682. doi: 10.3389/fphar.2024.1420682 (PMC11682906; doi:10.3389/fphar.2024.1420682)
Supplement: Supplementary file 4 [file Table2.docx]

| *Table S2. Costs by year and gram of non-specific immunoglobulins used in our calculations.* | | |
| --- | --- | --- |
| **Year of treatment** | **Type of immunoglobulin** | **Price (€) per gram of immunoglobulin** |
| 2019 | intravenous | 42,77 |
|  | subcutaneous | 42,33 |
| 2020 | intravenous | 42,33 |
|  | subcutaneous | 42,32 |
| 2021 | intravenous | 41,53 |
|  | subcutaneous | 42,36 |
