# Supplementary material for: Use of non-specific immunoglobulins in Catalonia in three third-level hospitals: a descriptive analysis of a hospital-prescribed medication registry
Source: Front Pharmacol. 2024 Dec 16;15:1420682. doi: 10.3389/fphar.2024.1420682 (PMC11682906; doi:10.3389/fphar.2024.1420682)
Supplement: Supplementary file 7 [file Table4.docx]

| *Table S4. Counts and percentages of indications by sex assigned at birth, hospital and level of evidence according to SISCAT guidelines.* | | | | | | | | | | | | | | | | | |  |  |  |
| --- | --- | --- | --- | --- | --- | --- | --- | --- | --- | --- | --- | --- | --- | --- | --- | --- | --- | --- | --- | --- |
| **ICD-10 Code** | **Indication** | **Indication subgroup** | **BUH** | | | | |  | **GTiPUH** | | | | | | **VHUH** | | | | | |
|  |  |  | Male | % | Female | % | All | % | Male | % | Female | % | All | % | Male | % | Female | % | All | % |
| **A - Authorised** | | | | | | | | | | | | | | | | | | | | |
| C90 | Replenishment therapy multiple myeloma with severe secondary hypogammaglobulinemia and recurrent infections | Multiple myeloma and malignant plasma cell neoplasms |  |  |  |  |  |  | 1 | 2.4 |  |  | 1 | 1.3 |  |  | 1 | 0.9 | 1 | 0.4 |
| C91 | Replenishment therapy chronic lymphatic leukemia with severe secondary hypogammaglobulinemia and recurrent infections | Lymphoid leukemia | 1 | 2.3 | 1 | 2.0 | 2 | 2.1 | 2 | 4.8 |  |  | 2 | 2.5 | 2 | 1.8 | 3 | 2.7 | 5 | 2.2 |
| D69 | Immunomodulation - Immune thrombocytopenic purpura (ITP) with impaired antibody production | Purpura and other hemorrhagic conditions | 1 | 2.3 | 1 | 2.0 | 2 | 2.1 | 1 | 2.4 | 1 | 2.6 | 2 | 2.5 | 4 | 3.5 | 9 | 8.0 | 13 | 5.8 |
| D69 | Immunomodulation - Primary immune thrombocytopenia with risk of bleeding | Purpura and other hemorrhagic conditions |  |  |  |  |  |  |  |  |  |  |  |  |  |  | 2 | 1.8 | 2 | 0.9 |
| D693 | Idiopathic thrombocytopenic purpura | Idiopathic thrombocytopenic purpura |  |  |  |  |  |  |  |  |  |  |  |  |  |  | 1 | 0.9 | 1 | 0.4 |
| D80 | Replenishment therapy in SIP - Congenital hypogammaglobulinemias | Immunodeficiency with predominantly antibody defects | 1 | 2.3 | 1 | 2.0 | 2 | 2.1 | 1 | 2.4 |  |  | 1 | 1.3 | 2 | 1.8 | 1 | 0.9 | 3 | 1.3 |
| D80 | Replenishment therapy in SIP - IgG subclass deficiency with recurrent infections | Immunodeficiency with predominantly antibody defects |  |  |  |  |  |  | 3 | 7.1 | 2 | 5.3 | 5 | 6.3 |  |  | 4 | 3.6 | 4 | 1.8 |
| D801 | Nonfamilial hypogammaglobulinemia | Nonfamilial hypogammaglobulinemia |  |  |  |  |  |  |  |  |  |  |  |  |  |  | 1 | 0.9 | 1 | 0.4 |
| D81 | Replenishment therapy in SIP - Combined severe immunodeficiency | Combined immunodeficiencies |  |  |  |  |  |  | 1 | 2.4 |  |  | 1 | 1.3 |  |  | 2 | 1.8 | 2 | 0.9 |
| D83 | Replacement therapy in SIP - Common variable immunodeficiency | Common variable immunodeficiency | 1 | 2.3 | 6 | 11.8 | 7 | 7.4 | 7 | 16.7 | 8 | 21.1 | 15 | 18.8 | 13 | 11.5 | 12 | 10.7 | 25 | 11.1 |
| D84 | Replacement therapy for primary antibody immunodeficiency syndrome (SIP) - non-specific | Other immunodeficiencies | 2 | 4.6 | 3 | 5.9 | 5 | 5.3 | 2 | 4.8 | 1 | 2.6 | 3 | 3.8 | 1 | 0.9 | 7 | 6.3 | 8 | 3.6 |
| G61 | Immunomodulation - Chronic inflammatory demyelinating polyneuropathy | Inflammatory polyneuropathy | 6 | 13.6 | 2 | 3.9 | 8 | 8.4 | 5 | 11.9 |  |  | 5 | 6.3 | 11 | 9.7 | 2 | 1.8 | 13 | 5.8 |
| G61 | Immunomodulation - Guillain Barré syndrome | Inflammatory polyneuropathy | 1 | 2.3 |  |  | 1 | 1.1 |  |  |  |  |  |  |  |  |  |  |  |  |
| G61 | Immunomodulation - Multifocal motor neuropathy | Inflammatory polyneuropathy | 11 | 25.0 | 3 | 5.9 | 14 | 14.7 |  |  |  |  |  |  |  |  | 3 | 2.7 | 3 | 1.3 |
| G6189 | Other inflammatory polyneuropathies | Other inflammatory polyneuropathies | 1 | 2.3 |  |  | 1 | 1.1 |  |  |  |  |  |  |  |  |  |  |  |  |
| G70 | Myasthenia gravis | Myasthenia gravis and other myoneural disorders | 7 | 15.9 | 12 | 23.5 | 19 | 20.0 | 3 | 7.1 | 4 | 10.5 | 7 | 8.8 | 3 | 2.7 | 9 | 8.0 | 12 | 5.3 |
| G7000 | Myasthenia gravis without (acute) exacerbation | Myasthenia gravis without (acute) exacerbation |  |  |  |  |  |  |  |  |  |  |  |  | 1 | 0.9 |  |  | 1 | 0.4 |
| T8691 | Antibody-mediated rejection treatment in solid organ transplant | Unspecified transplanted organ and tissue rejection | 5 | 11.4 | 2 | 3.9 | 7 | 7.4 | 3 | 7.1 | 6 | 15.8 | 9 | 11.3 | 6 | 5.3 | 6 | 5.4 | 12 | 5.3 |
| Z94 | Replenishment therapy before and after allogeneic hematopoietic stem cell transplantation with hypogammaglobulinemia | Stem cells transplant status |  |  |  |  |  |  |  |  |  |  |  |  | 1 | 0.9 |  |  | 1 | 0.4 |
| Z94 | Desensitizer treatment before a high-risk immunological solid organ transplant | Transplanted organ and tissue status |  |  |  |  |  |  |  |  |  |  |  |  | 3 | 2.7 | 1 | 0.9 | 4 | 1.8 |
| Z94 | Desensitizer treatment before an ABO-incompatible or HLA-incompatible transplant | Transplanted organ and tissue status |  |  |  |  |  |  |  |  |  |  |  |  | 2 | 1.8 |  |  | 2 | 0.9 |
| Z940 | Kidney transplant status | Kidney transplant status |  |  |  |  |  |  | 1 | 2.4 |  |  | 1 | 1.3 | 10 | 8.9 | 1 | 0.9 | 11 | 4.9 |
| Z944 | Liver transplant status | Liver transplant status |  |  |  |  |  |  |  |  |  |  |  |  |  |  | 2 | 1.8 | 2 | 0.9 |
| Z9481 | Bone marrow transplant status | Bone marrow transplant status |  |  |  |  |  |  |  |  |  |  |  |  | 3 | 2.7 |  |  | 3 | 1.3 |
| Z9484 | Replenishment therapy before and after allogeneic hematopoietic stem cell transplantation with hypogammaglobulinemia | Stem cells transplant status |  |  |  |  |  |  | 1 | 2.4 |  |  | 1 | 1.3 | 17 | 15.0 | 6 | 5.4 | 23 | 10.2 |
| **TOTAL A** | |  | **37** | **84.1** | **31** | **60.8** | **68** | **71.6** | **31** | **73.8** | **22** | **57.9** | **53** | **66.3** | **79** | **69.9** | **73** | **65.2** | **152** | **67.5** |
| **B - Not authorised with evidence** | | | | | | | | | | | | | | | | | | | | |
| D82 | Replenishment therapy for secondary antibody deficiency syndrome | Immunodeficiency associated with other major defects |  |  | 4 | 7.8 | 3 | 3.2 | 1 | 2.4 | 3 | 7.9 | 4 | 5.0 | 10 | 8.9 | 9 | 8.0 | 19 | 8.4 |
| D82 | Secondary immunodeficiency replacement therapy with severe or recurrent infections, ineffective antimicrobial treatment and proven failure of specific antibodies (PSAF) | Immunodeficiency associated with other major defects | 1 | 2.3 | 2 | 3.9 | 4 | 4.2 |  |  | 1 | 2.6 | 1 | 1.3 | 5 | 4.4 | 4 | 3.6 | 9 | 4.0 |
| G04 | Autoimmune or paraneoplastic encephalitis | Encephalitis, myelitis and encephalomyelitis |  |  |  |  |  |  | 2 | 4.8 | 4 | 10.5 | 6 | 7.5 | 2 | 1.8 | 3 | 2.7 | 5 | 2.2 |
| G25 | Stiff person syndrome | Other extrapyramidal and movement disorders |  |  | 1 | 2.0 | 1 | 1.1 |  |  |  |  |  |  |  |  | 1 | 0.9 | 1 | 0.4 |
| G40 | Severe and refractory epilepsy | Epilepsy |  |  |  |  |  |  |  |  | 1 | 2.6 | 1 | 1.3 | 1 | 0.9 |  |  | 1 | 0.4 |
| G70 | Lambert-Eaton myasthemic syndrome | Myasthenia gravis and other myoneural disorders | 2 | 4.6 | 4 | 7.8 | 6 | 6.3 | 1 | 2.4 |  |  | 1 | 1.3 |  |  |  |  |  |  |
| G7249 | Other inflammatory and immune myopathies, not elsewhere classified | Other inflammatory and immune myopathies, not elsewhere classified |  |  |  |  |  |  |  |  |  |  |  |  | 2 | 1.8 |  |  | 2 | 0.9 |
| G729 | Myopathy, unspecified | Myopathy, unspecified | 2 | 4.6 |  |  | 2 | 2.1 |  |  |  |  |  |  |  |  | 2 | 1.8 | 2 | 0.9 |
| I77 | Replacement therapy in ANCA vasculitis | Other disorders of arteries and arterioles |  |  | 1 | 2.0 | 1 | 1.1 |  |  | 1 | 2.6 | 1 | 1.3 |  |  | 1 | 0.9 | 1 | 0.4 |
| M32 | Systemic lupus erythematosus (SLE) | Systemic lupus erythematosus (SLE) |  |  |  |  |  |  |  |  |  |  |  |  |  |  | 3 | 2.7 | 3 | 1.3 |
| M3302 | Juvenile dermatomyositis with myopathy | Juvenile dermatomyositis with myopathy |  |  |  |  |  |  |  |  |  |  |  |  |  |  | 1 | 0.9 | 1 | 0.4 |
| M3322 | Polymyositis with myopathy | Polymyositis with myopathy |  |  | 1 | 2.0 | 1 | 1.1 |  |  |  |  |  |  | 1 | 0.9 |  |  | 1 | 0.4 |
| M3390 | Dermatopolymyositis, unspecified, organ involvement unspecified | Dermatopolymyositis, unspecified, organ involvement unspecified | 1 | 2.3 | 1 | 2.0 | 2 | 2.1 | 2 | 4.8 | 3 | 7.9 | 5 | 6.3 | 1 | 0.9 | 3 | 2.7 | 4 | 1.8 |
| M3391 | Dermatopolymyositis, unspecified with respiratory involvement | Dermatopolymyositis, unspecified with respiratory involvement |  |  |  |  |  |  |  |  |  |  |  |  | 1 | 0.9 |  |  | 1 | 0.4 |
| M360 | Dermato(poly)myositis in neoplastic disease | Dermato(poly)myositis in neoplastic disease |  |  |  |  |  |  |  |  | 1 | 2.6 | 1 | 1.3 |  |  |  |  |  |  |
| Z23 | Need for immunization against single bacterial diseases in the context of liver transplantation | Need for immunization against single bacterial diseases | 1 | 2.3 |  |  | 1 | 1.1 |  |  |  |  |  |  |  |  |  |  |  |  |
| **TOTAL B** | |  | **7** | **15.9** | **14** | **27.4** | **21** | **22.1** | **6** | **14.3** | **14** | **36.8** | **20** | **25.0** | **23** | **20.3** | **27** | **24.1** | **50** | **22.2** |
| **C - Not authorised without evidence** | | | | | | | | | | | | | | | | | | | | |
| D599 | Acquired hemolytic anemia, unspecified | Acquired hemolytic anemia, unspecified |  |  | 1 | 2.0 | 1 | 1.1 |  |  |  |  |  |  |  |  |  |  |  |  |
| D849 | Immunodeficiency, unspecified | Immunodeficiency, unspecified |  |  |  |  |  |  |  |  |  |  |  |  |  |  | 1 | 0.9 | 1 | 0.4 |
| G61 | Demyelinating neuropathy associated with paraproteins (IgM) | Inflammatory polyneuropathy |  |  | 1 | 2.0 | 1 | 1.1 | 1 | 2.4 |  |  | 1 | 1.3 |  |  |  |  |  |  |
| **TOTAL C** | |  | **0** | **0.0** | **2** | **3.9** | **2** | **2.1** | **1** | **2.4** | **0** | **0.0** | **1** | **1.3** | **0** | **0.0** | **1** | **0.9** | **1** | **0.4** |
| **UNK - Unknown/Not classified** | | | | | | | | | | | | | | | | | | | | |
| A430 | Pulmonary nocardiosis | Pulmonary nocardiosis |  |  |  |  |  |  |  |  |  |  |  |  | 1 | 0.9 |  |  | 1 | 0.4 |
| B349 | Viral infection, unspecified | Viral infection, unspecified |  |  | 1 | 2.0 | 1 | 1.1 |  |  |  |  |  |  |  |  |  |  |  |  |
| C8580 | Other specified types of non-Hodgkin lymphoma, unspecified site | Other specified types of non-Hodgkin lymphoma, unspecified site |  |  |  |  |  |  |  |  |  |  |  |  | 1 | 0.9 |  |  | 1 | 0.4 |
| C9102 | Acute lymphoblastic leukemia, in relapse | Acute lymphoblastic leukemia, in relapse |  |  |  |  |  |  |  |  |  |  |  |  | 1 | 0.9 |  |  | 1 | 0.4 |
| D68312 | Antiphospholipid antibodies with hemorrhagic disorder | Antiphospholipid antibody with hemorrhagic disorder |  |  |  |  |  |  |  |  |  |  |  |  | 1 | 0.9 |  |  | 1 | 0.4 |
| D891 | Cryoglobulinemia | Cryoglobulinemia |  |  |  |  |  |  |  |  |  |  |  |  |  |  | 1 | 0.9 | 1 | 0.4 |
| D8982 | Autoimmune lymphoproliferative syndrome [SLPA] | Autoimmune lymphoproliferative syndrome [ALPS] |  |  | 1 | 2.0 | 1 | 1.1 |  |  |  |  |  |  |  |  |  |  |  |  |
| G36 | Other acute disseminated demyelination | Other acute disseminated demyelination |  |  |  |  |  |  |  |  |  |  |  |  |  |  | 1 | 0.9 | 1 | 0.4 |
| G629 | Unspecified polyneuropathy | Polyneuropathy, unspecified |  |  |  |  |  |  | 1 | 2.4 |  |  | 1 | 1.3 |  |  |  |  |  |  |
| G712 | Congenital myopathies | Congenital myopathies |  |  | 1 | 2.0 | 1 | 1.1 |  |  |  |  |  |  |  |  |  |  |  |  |
| I6350 | Cerebral infarction due to unspecified occlusion or stenosis of unspecified cerebral artery | Cerebral infarction due to unspecified occlusion or stenosis of unspecified cerebral artery |  |  |  |  |  |  | 1 | 2.4 |  |  | 1 | 1.3 |  |  |  |  |  |  |
| I639 | Cerebral infarction, unspecified | Cerebral infarction, unspecified |  |  |  |  |  |  |  |  |  |  |  |  | 1 | 0.9 |  |  | 1 | 0.4 |
| I677 | Cerebral arteritis, not elsewhere classified | Cerebral arteritis, not elsewhere classified |  |  |  |  |  |  |  |  |  |  |  |  |  |  | 1 | 0.9 | 1 | 0.4 |
| L0100 | Impetigo, unspecified | Impetigo, unspecified |  |  |  |  |  |  |  |  |  |  |  |  | 1 | 0.9 |  |  | 1 | 0.4 |
| L959 | Vasculitis limited to the skin, unspecified | Vasculitis limited to the skin, unspecified |  |  |  |  |  |  |  |  |  |  |  |  | 1 | 0.9 |  |  | 1 | 0.4 |
| M048 | Other autoinflammatory syndromes | Other autoinflammatory syndromes |  |  |  |  |  |  |  |  |  |  |  |  | 1 | 0.9 |  |  | 1 | 0.4 |
| M340 | Progressive systemic sclerosis | Progressive systemic sclerosis |  |  |  |  |  |  | 1 | 2.4 |  |  | 1 | 1.3 |  |  | 1 | 0.9 | 1 | 0.4 |
| M349 | Systemic sclerosis, unspecified | Systemic sclerosis, unspecified |  |  |  |  |  |  |  |  |  |  |  |  | 1 | 0.9 |  |  | 1 | 0.4 |
| M3500 | Sjögren syndrome, unspecified | Sjögren syndrome, unspecified |  |  |  |  |  |  |  |  | 1 | 2.6 | 1 | 1.3 |  |  |  |  |  |  |
| M459 | Ankylosing spondylitis of unspecified sites in spine | Ankylosing spondylitis of unspecified sites in spine |  |  |  |  |  |  |  |  |  |  |  |  |  |  | 1 | 0.9 | 1 | 0.4 |
| M609 | Myositis, unspecified | Myositis, unspecified |  |  |  |  |  |  | 1 | 2.4 |  |  | 1 | 1.3 |  |  |  |  |  |  |
| N158 | Other specified renal tubulo-interstitial diseases | Other specified renal tubulo-interstitial diseases |  |  | 1 | 2.0 | 1 | 1.1 |  |  |  |  |  |  |  |  |  |  |  |  |
| N186 | End stage renal disease | End stage renal disease |  |  |  |  |  |  |  |  |  |  |  |  | 1 | 0.9 |  |  | 1 | 0.4 |
| O09299 | Supervision of pregnancy with other poor reproductive or obstetric history, unspecified trimester | Supervision of pregnancy with other poor reproductive or obstetric history, unspecified trimester |  |  |  |  |  |  |  |  |  |  |  |  |  |  | 1 | 0.9 | 1 | 0.4 |
| O80 | Singleton spontaneous delivery | Singleton spontaneous delivery |  |  |  |  |  |  |  |  |  |  |  |  |  |  | 2 | 1.8 | 2 | 0.9 |
| R0600 | Dyspnea, unspecified | Dyspnea, unspecified |  |  |  |  |  |  |  |  | 1 | 2.6 | 1 | 1.3 |  |  |  |  |  |  |
| R180 | Malignant ascites | Malignant ascites |  |  |  |  |  |  |  |  |  |  |  |  | 1 | 0.9 |  |  | 1 | 0.4 |
| R69 | Unknown and unspecified causes of morbidity | Unknown and unspecified causes of morbidity |  |  |  |  |  |  |  |  |  |  |  |  |  |  | 1 | 0.9 | 1 | 0.4 |
| S27301S | Unspecified injury of lung, unilateral | Unspecified injury of lung, unilateral |  |  |  |  |  |  |  |  |  |  |  |  |  |  | 1 | 0.9 | 1 | 0.4 |
| Z8279 | Family history of other congenital malformations, deformations and chromosomal abnormalities | Family history of other congenital malformations, deformations and chromosomal abnormalities |  |  |  |  |  |  |  |  |  |  |  |  |  |  | 1 | 0.9 | 1 | 0.4 |
| **TOTAL UNKNOWN** | |  | **0** | **0.0** | **4** | **7.8** | **4** | **4.2** | **4** | **9.5** | **2** | **5.3** | **6** | **7.5** | **11** | **9.7** | **11** | **9.8** | **22** | **9.7** |
| **TOTAL SAMPLE (N=400)** | |  | **44** | **100.0** | **51** | **100.0** | **95** | **100.0** | **42** | **100.0** | **38** | **100.0** | **80** | **100.0** | **113** | **99.9** | **112** | **100.0** | **225** | **100** |
| *BUH: Bellvitge University Hospital; GTiPUH: Germans Trias i Pujol University Hospital; ICD10: International Classification of Diseases, Tenth Revision; SISCAT: Public Catalan Health System; VHUH: Vall d'Hebron University Hospital* | | | | | | | | | | | | | | | | | | | | |
