# Supplementary material for: Use of non-specific immunoglobulins in Catalonia in three third-level hospitals: a descriptive analysis of a hospital-prescribed medication registry
Source: Front Pharmacol. 2024 Dec 16;15:1420682. doi: 10.3389/fphar.2024.1420682 (PMC11682906; doi:10.3389/fphar.2024.1420682)
Supplement: Supplementary file 8 [file Table6.docx]

| *Table S6. Discontinuation counts and percentages by hospital and level of evidence according to SISCAT guidelines.* | | | | | | | | | |
| --- | --- | --- | --- | --- | --- | --- | --- | --- | --- |
|  | **BUH, n(%)** | | | **GTiPUH, n(%)** | | | **VHUH, n(%)** | | |
| **Level of evidence** | **Discontinuers** | **Non-discontinuers** | **Total** | **Discontinuers** | **Non-discontinuers** | **Total** | **Discontinuers** | **Non-discontinuers** | **Total** |
| ***Adults (≥18 years of age)*** | | | | | | | | | |
| **A-level** | 28 (41.2) | 40 (58.8) | 68 (71.6) | 8 (16.0) | 42 (84.0) | 50 (64.9) | 44 (44.4) | 55 (55.6) | 99 (62.3) |
| **B-level** | 3 (14.3) | 18 (85.7) | 21 (22.1) | 5 (25.0) | 15 (75.0) | 20 (26.0) | 16 (37.2) | 27 (62.8) | 43 (27.0) |
| **C-level** | 2 (100) | 0 (0) | 2 (2.1) | 0 (0) | 1 (100) | 1 (1.3) | 1 (100) | 0 (0) | 1 (0.6) |
| **UNK-level** | 1 (25.0) | 3 (75.0) | 4 (4.2) | 2 (33.3) | 4 (66.7) | 6 (6.3) | 6 (37.5) | 10 (62.5) | 16 (10.1) |
| **TOTAL** | 34 (35.8) | 61 (64.2) | 95 (100) | 15 (19.5) | 62 (80.5) | 77 (100) | 67 (42.1) | 92 (57.9) | 159 (100) |
|  | | | | | | | | |  |
| ***Pediatrics (<18 years of age)*** | | | | | | | | | |
| **A-level** |  |  |  | 3 (100) | 0 (0) | 3 (100) | 26 (49.1) | 27 (50.9) | 53 (80.3) |
| **B-level** |  |  |  |  |  |  | 2 (28.6) | 5 (71.4) | 7 (10.6) |
| **C-level** |  |  |  |  |  |  | 0 (0) | 0 (0) | 0 (0) |
| **UNK-level** |  |  |  |  |  |  | 4 (66.7) | 2 (33.3) | 6 (9.1) |
| **TOTAL** |  |  |  | 3 (100) | 0 (0) | 3 (100) | 32 (48.5) | 34 (51.5) | 66 (100) |
| *BUH: Bellvitge University Hospital; GTiPUH: Germans Trias i Pujol University Hospital; SISCAT: Public Catalan Health System; VHUH: Vall d'Hebron University Hospital* | | | | | | | | | |
